# Effect of Dietary Grapes on Female C57BL6/J Mice Consuming a High-Fat Diet: Behavioral and Genetic Changes

**DOI:** 10.3390/antiox11020414

**Published:** 2022-02-18

**Authors:** Falguni Parande, Asim Dave, Eun-Jung Park, Christopher McAllister, John M. Pezzuto

**Affiliations:** 1 Arnold and Marie Schwartz College of Pharmacy and Health Sciences, Long Island University, New York, NY 11201, USA; falguni.parande@my.liu.edu (F.P.); asim.dave@my.liu.edu (A.D.); eunjung.park@liu.edu (E.-J.P.); 2 College of Veterinary Medicine, Long Island University, New York, NY 11548, USA; christopher.mcallister@liu.edu; 3 College of Pharmacy and Health Sciences, Western New England University, Springfield, MA 01108, USA

**Keywords:** high-fat diet, mouse model, anxiety, cognition, RNA-Seq, dopamine activity, eating behavior, open field test, novel object recognition test, radial arm maze, nutrigenomics

## Abstract

(1) Background: Adverse effects of a chronic high-fat diet (HFD) on murine behavior, cognition, and memory are well established. Polyphenols such as resveratrol, anthocyanins, and flavonoids, that are known for antioxidative and anti-inflammatory properties, are present in grapes. The objective of this work was to determine if the dietary intake of grapes has the potential of alleviating HFD-induced deficiencies. (2) Methods: The effect of dietary grape intake was studied using behavioral assays and high throughput genome-wide RNA transcriptome analyses with female C57BL6/J mice. (3) Results: Mice that were fed a HFD from 3-weeks of age showed anxiety-like behaviors compared with the standard diet (STD). This HFD-induced effect was attenuated by supplementing the HFD with 1% grape powder (HF1G) (open field test). Similar results were observed with the novel object recognition test; there was a significant difference in time spent exploring a novel object between the HFD and the HF1G groups. There was no significant difference between the HFD1G and the STD groups. Based on the RNA-Seq analysis, genetic expression in the brain varied as a result of diet, with 210, 360, and 221 uniquely expressed genes in the STD, HFD, and HF1G groups, respectively. Cluster analysis revealed that the HFIG group mapped more closely with the STD group than the HFD group. Focusing on some specific areas, based on genetic expression, Dopamine receptor 2 (Drd2) was increased in the HFD group and normalized in the HF1G group, relative to the STD group. In addition, as judged by cluster hierarchy, the expression of genes that are associated with the dopamine receptor 2 pathway were increased in the HFD group, whereas the pattern that was derived from mouse brain from the HF1G group showed greater similarity to the STD group. KEGG pathway analyses were consistent with these results. For example, neuroactive ligand-receptor interaction (KEGG ID: mmu04080) was altered due to HFD compared with STD, but normalized by grape supplementation or the HFD; there was no significant difference between the STD and HF1G groups. In addition, the expression of genes related to feeding behavior, such as Adora2a, Th, and Trh, were also increased in the HFD group compared with the STD group, and attenuated by grape supplementation. (4) Conclusions: Dietary grape consumption has positive effects on behavior and cognition that are impaired by a HFD. Attenuation of these effects correlates with global transcriptional changes in mouse brain.

## 1. Introduction

Obesity is one of the leading concerns in the healthcare sector since it is a gateway to metabolic syndrome. In addition to genetic background, various factors may contribute to metabolic syndromes, such as lifestyle choices and environmental factors. In the U.S., obesity is the most prevalent health condition; 42.4% of the population face obesity-related conditions such as heart disease, type 2 diabetes, hypertension, and certain types of cancer [1].

Besides these pathophysiological changes, mental and behavioral concerns are as-sociated with high-fat diet (HFD) consumption (a.k.a. Western pattern diet). HFD consumption alters neurochemistry and can be responsible for physiological responses that lead to oxidative stress and neuroinflammatory diseases. There are also minute structural changes to the neuroanatomy of the brain. These effects are manifested with depression and anxiety-like behaviors or impaired cognition and/or memory deficits [2]. Epidemiological studies have shown that emotional disturbances such as anxiety and depression are associated with chronic consumption of a HFD [1]. Further, the neurobiology of depression alone causes anhedonia, changes in feeding behavior and food-seeking behavior; consumption of HFD aggravates the condition [2].

Spearheaded by a conceptualization of the “French Paradox” [3], the potential health benefits of grapes [4] and the phenolic constituents that are present in wine and grapes [5], have been widely studied, including the neuroprotective effects of grape consumption. Grapes contain numerous polyphenolic compounds such as resveratrol, anthocyanin, proanthocyanin, ellagic acid, etc. These polyphenolic compounds possess several biological activities such as antioxidant, anti-inflammatory, and antiaging that are proven to be beneficial for overall health [5]. Grapes have been known to have a certain potential benefits for Alzheimer’s disease and other neurodegenerative diseases [6]. For example, there is clinical evidence showing the neuroprotective effects of grapes in maintaining better mental health and cognitive ability [7].

In the current study, we attempted to provide some rudimentary data relating to diet and brain function by limiting variables and focusing on two key modulating factors: the high-fat (Western pattern) diet, known to cause ill-health effects, and the potential of dietary grapes (known to contain over 1600 phytochemicals) [8], to ameliorate high-fat diet-induced responses. The potential impact of grapes and grape products such as grape juice, grape seeds, and wine, to influence various aspects of health have been widely investigated [4,8], including brain function [9]. Promising results have been reported but open questions regarding the scope of activity and the mechanism of action remain open. Accordingly, we elected to continue in the area of grape research. It is also noteworthy that the continuity of research is facilitated by the availability of a standardized product that is subjected to quality control measures.

As reported herein, we examined the effect of dietary intake of HFD that was supplemented with 1% grape powder (HF1G), relative to a control HFD, and a standard diet (STD), in the female C57BL6/J mouse brain, using behavioral studies and high throughput RNA-sequencing.

## 2. Materials and Methods

### 2.1. Diets and Freeze-Dried Grape Powder

To assure the consistency and continuity of experimental and clinical research concerning the biological and physiologic potential of grapes, a freeze-dried powder is available that serves as a surrogate for fresh grapes. The grape powder is composed of fresh seeded and seedless red, green, and black grapes that are freeze-dried and grounded to retain their bioactive compounds. The standard product is subjected to chemical and microbial analyses to assure quality [10].

For the current studies, standardized freeze-dried grape powder [10] was supplied in vacuum-sealed packets by the California Table Grape Commission (Fresno, CA, USA) and stored at −20 °C. Based on the composition of grapes, isocaloric diets were custom-designed and produced by Envigo (Madison, WI, USA) (Table 1).

### 2.2. Animals and Feeding

A total of 65 three-week-old C57BL/6J female mice were purchased from Jackson Laboratory (Bar Harbor, ME). The mice were housed in ventilated cages with standard room temperature and humidity control with a 12-h light-dark cycle. The mice were provided with STD and water ad libitum. At the eleventh week, the mice were randomly divided into three diet groups and provided with STD, HFD, or HF1G (Table 1), and water ad libitum. The body weight of each mouse was determined every second week. A total of five mice from each group were euthanized at 6 months for RNA-Seq analysis.

For the purpose of these studies, the determination of estrus cycles was not deemed as necessary. The mice were housed in a room that was limited to only females. No male mice were in the room or in any close proximity. Moreover, it has been reported in the literature that the behavior of C57BL/6J females remains stable across all four phases of the estrous cycle in tests including open field, rotarod, startle reflex and pre-pulse inhibition, tail flick, and hot plate [11]. Further, given the nature of our results, it is reasonable to assume that either the mice were cycling in a synchronous manner due to the housing conditions, or the phase of the cycle did not lead to variability, as described previously [11].

This study was conducted in accordance with the IACUC protocol that was approved at Long Island University (protocol number 19-07).

### 2.3. Behavioral Tests

A total of 30 6-month-old mice were included in the open field test to study anxiety. Age-associated memory and cognitive decline were investigated when the mice were 18-months-old using the novel object recognition and radial arm maze tests. These studies were conducted in a controlled environment (sound: 50 dB; light: 15 lux; temperature: 22.5 °C; humidity: 28%).

#### 2.3.1. Open Field Test

One designated person handled the mice for 3 to 4 days before the day of conducting the experiment. The mice were brought into the room 1 h before the experiment to acclimatize to the environment. The open field test entailed a two-day protocol. On the first day, each mouse was placed in the open field for 10 min. On the test day, the mice were placed in the center of the field and allowed to explore the area. The movement of the mice was tracked and recorded with a camera that was placed over the field and analyzed using Anymaze software (Stoelting Co., Wood Dale, IL, USA).

#### 2.3.2. Novel Object Recognition Test (NORT)

The novel object recognition test was a 3-day process. The experiment was conducted on a 100 × 100 cm^2^ open field with dividers that separate the open field into four quadrants making each quadrant a 50 × 50 cm^2^ open field, with a video camera mounted directly above the open field to record movement. On the first and second day, the mice were habituated to the open field for 10 min. On the third day, the mice were trained with two identical building blocks that were kept in the center of the open field, placed 20 cm away from the walls horizontally and vertically. Familiarization with the objects was allowed for 10 min for each mouse. After 12 h of retention, the mice were placed in the field with the objects only this time, with one of the familiarized objects being replaced with a novel object (owl figurine). The open field had four quadrants; therefore, a set of four mice were tested at one time. The entire field was recorded and analyzed with Any-Maze Software (Stoelting Co., IL, USA). The time that the mice took for exploring each zone was monitored and the discrimination index score was calculated to quantify cognition (below). This assay was conducted with mice that were 18 months of age.

$$Discrimination\;Index=\frac{\left(time\;spent\;on\;novel\;object-time\;spent\;on\;familiar\;object\right)}{Time\;spent\;on\;both\;the\;objects}$$

#### 2.3.3. Radial Arm Maze

The radial arm maze test was a 5-day process. The experiment was conducted with an eight-armed commercially available radial arm maze with a center platform (MazeEngineers, Skokie, IL, USA). The mice were habituated to the radial arm maze for two days for 10 min per day, and then they were placed in the center of the maze and allowed to roam each arm freely. On the third day, baits (food pellets from their respective diet) were placed in a well at the end of each arm to promote exploration. On the fourth day, the number of baited arms was reduced to half, and the session ended when all eight arms were visited (mice were considered to have entered an arm of the maze if the whole body was in the arm except for the tail) or 10 min after the start of the session. On the fifth day, a similar trial was conducted to observe if the mouse remembered to enter the arms that were baited during training. The maze was wiped clean with 70% ethanol between each trial to prevent any odor cues. The mouse activity in the entire maze was recorded and analyzed with Any-Maze Software (Stoelting Co., Wood Dale, IL, USA). Variables commonly that are used for the analysis of the performance are working memory error and reference memory error. Re-entry in a previously visited arm is counted as a working memory error, and entry in a non-baited arm is considered a reference memory error.

### 2.4. Brain Tissue Collection

At approximately 6 months of age, five mice from each diet group were randomly selected for sacrifice one week after completing all the behavioral studies. The mice were fasted for 12 h (10 PM to 10 AM) to avoid circadian disruptions. The mice were placed in a CO_2_ chamber for two to three minutes. The brain tissue from each individual mouse was isolated immediately to prevent degradation. The tissue samples were snap-frozen in liquid nitrogen and stored at −80 °C until shipment to Novogene (Sacramento, CA, USA) for RNA extraction and sequencing.

### 2.5. RNA-Extraction and Sequencing

The process of RNA extraction and sequencing was performed by Novogene (Sacramento, CA, USA) with each individual mouse brain (five separate tissues/group). RNA was quantified to prepare the transcriptome library. High-quality RNA (i.e., samples achieving an RNA integrity number of >6) was subjected to mRNA purification using magnetic beads, and cDNA was synthesized as per the script sequencing protocol. The RNA-integrity was higher for 4 samples/group. After the library was checked for quality using a Qubit and RT-PCR for quantification, the quantified libraries were sequenced. The amount of transcriptome directly translates to the expression of the specific genes.

### 2.6. Differentially Expressed Genes

The RNA-sequencing was outsourced to Novogene (Sacramento, CA, USA) for original data processing and related bioinformatics analysis with the frozen brain tissue samples. The differential expression of genes was analyzed between two diet groups. All possible permutations were used to analyze and compare all the diet groups. A total of five brain tissues that were derived from five individual mice in each group were processed. One from each group did not attain a sufficient RNA integrity number (<6). Thus, four individual samples from each diet group were subjected to complete analysis. Data from each group were combined for graphical presentation and comparisons between the groups, which was done using the DESeq2 R package (1.14.1). It provided custom statistical analysis for determining the differences in expression between the digital gene expression data of two groups using a model that was based on the negative binomial distribution. The culminating *p*-values were controlled using Benjamini and Hochberg’s approach for false discovery rate (FDR). The differentially expressed genes (with a *p* < 0.05) were uncovered by DESeq2 (for edgeR without biological replicates). Before differential gene expression analysis, the read counts were adjusted by the edgeR program package for each sequenced library through one scaling normalized factor. Differential expression analysis of two conditions was performed using the edgeR R package (3.16.5)

Corrected *p* < 0.05 and absolute log2 fold-change that were higher than 1 or lower than −1 was set as the threshold for significantly differential expression. R Program was used to prepare the Venn diagrams using the function Venn Diagram on the gene list from the different diet groups. The programs that were used for the downstream analysis included STAR, HTseq, Cufflink, and our wrapped scripts. The alignments were parsed using the STAR program, and differential expression was determined through DESeq2/edgeR, GO, and the Cluster Profiler implemented KEGG enrichment. Star-fusion and rMATS software detected gene fusion and differences of alternative splicing events.

### 2.7. Gene Ontology

Gene ontology (GO) (http://geneontology.org/ accessed on 20 September 2021) and PANTHER classification system (http://pantherdb.org/ accessed on 20 September 2021) are the web tools that were used to process the differential gene expression (DEG) list to envision any relevant biological changes upon exposure to HFD and the alterations that were brought by grape intervention. The data mining of these web tools is used for obtaining the pathways for the molecular changes, such as signaling pathways and the protein sequences mapped for components that are involved in those pathways. The functional annotations that relate a particular gene with a GO term which constitutes of biological domains such biological processes, cellular components, and molecular functions. These GO terms give a detailed explanation of the role of a particular genetic product in a biological process, and the molecular activities that are involved in those processes as well as the cellular components and the cell organelles (e.g., ribosome or mitochondria) that are involved in those molecular activities.

### 2.8. KEGG Pathway Analyses

Kyoto Encyclopedia of Genes and Genomes (KEGG) is a database of signaling pathways that includes a network of genes that are involved in signaling pathways. To provide a better understanding of biological system alteration at a molecular level due to consumption of HFD or HF1G, Cluster profiler (R package) was used to generate a list of signaling pathways from a subset of genes (differentially-expressed genes between two diet groups) that overlap with those in the database (http://www.kegg.jp/ accessed on 20 September 2021). Significance was determined with FDR values < 0.05.

### 2.9. Statistical Analyses

Statistical analysis was conducted using Microsoft^®^ Excel and GraphPad Prism 9. The statistical analysis, as noted throughout the text, were conducted using ANOVA, Pearson correlation test, Student’s *t*-test assuming equal differences, and paired *t*-test, two samples for means. R-studio was used to perform the cluster analysis and create a heatmap. After removing the outliers in all the experiments, the data were analyzed, which were calculated using a graph-pad outlier calculator (https://www.graphpad.com/quickcalcs/Grubbs1.cfm accessed on 8 August 2021). To identify the correlation in differentially-expressed genes among the different diet groups, FPKM values were normalized and the hierarchical clustering method was used to generate heat maps to illustrate the data. Heatmaps were generated using the online web tool DisplayR (app.displayr.com accessed on 8 August 2021). The results are expressed as a mean ± SD and *p* < 0.05 was taken to indicate statistical significance.

## 3. Results

### 3.1. Animal Body Weight

**Figure 1:**
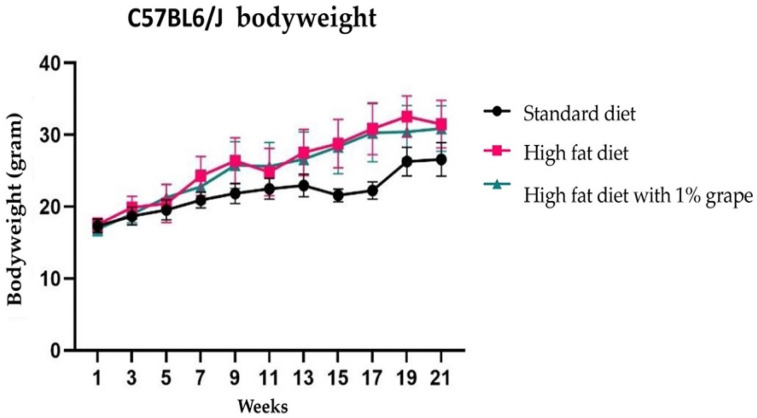
Body weight of C57BL6/J female mice following placement on the indicated diets.

As shown in Figure 1, after 21 weeks on the respective diets, there was an increase in the body weight of mice that were provided with the HFD (30.23 ± 3.20 g) and HF1G (31.31 ± 6.1 g) compared with those that were provided with the STD (26.59 ± 2.34 g) (two-way ANOVA F (2, 42) = 15.13, *p* = 0.00). Further Dunnett’s multiple comparison revealed that there was no significant difference between the HFD and HF1G groups (*p* = 0.83). As described previously, the addition of grape powder to murine diets does not affect the overall rate of consumption [12].

### 3.2. Behavioral Assays

#### 3.2.1. Open Field Test

**Figure 2:**
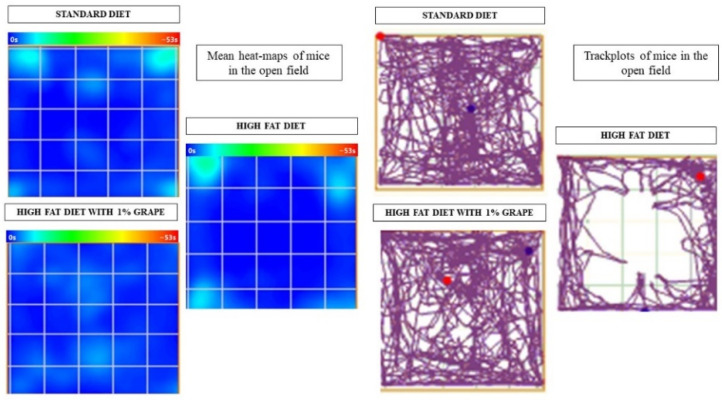
Heatmaps and track plots of mice that were placed on the open field to observe anxiety-like behavior. Mice that were placed on HF1G diet spent more time in the center (203.4 ± 94.9 s) as compared to the HFD control group (90.3 ± 86.4 s) [two-way ANOVA: *F* (2, 25) = 4.33, *p* = 0.02]. Conversely, mice that were placed on the HF1G diet spent significantly less time in the corners (156.6 ± 61.6 s) as compared to the HFD group (279.3 ± 105.6 s) [two-way ANOVA: *F* (2, 25) = 3.96, *p* = 0.04]. There was no significant difference that was observed between the STD (269.5 ± 149.4 s) and HF1G groups (Student’s *t*-test, *p* = 0.60, *n* = 30).

The tendency to remain close to the open field apparatus walls indicates the anxiety-like behavior of the mice. Therefore, the time spent in the corner zone indicates anxiety-like behavior, while the time spent in the center area indicates exploratory behavior. The accumulative visual results that were obtained using Anymaze software and displayed as heatmaps and track plots are shown in Figure 2.

**Figure 3:**
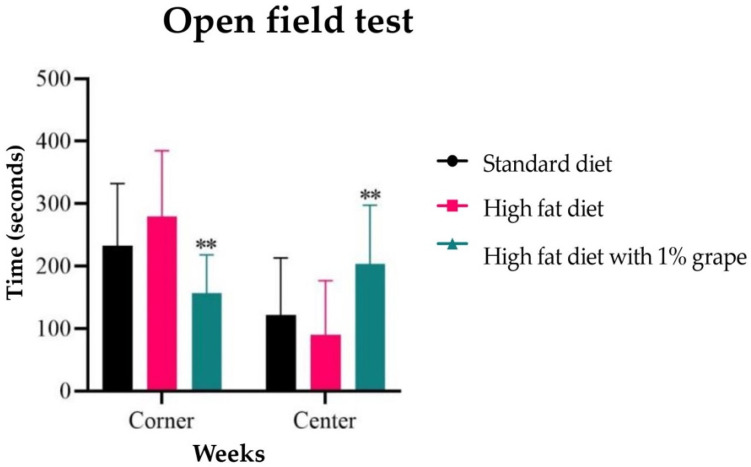
Open field test -The time spent in the corners and center of the field by mice that were placed on different types of diets. Mice that were provided with the HFD spend more time in corners than the center arena of the open field, indicating anxious behavior, while by comparison, mice that were provided with the HFD1G showed more exploratory behavior. The time spent in corner zone: ** STD vs. HFD and HFD vs. HF1G, *p* < 0.01; HF1G vs. STD *p* = 0.05. Time spent in center zone: HFD vs. HF1G, *p* = 0.01; STD vs. HFD, *p* = 0.6; STD vs. HF1G, *p* = 0.05 (Student’s *t*-test, *n* = 30) (bars represent standard deviation, *p* < 0.05, ** *p* < 0.01).

Quantitative analyses of the data are presented in Figure 3. Mice that were placed on the HF1G diet spent more time in the center (203.4 ± 94.9 s) as compared to the HFD control group (90.3 ± 86.4 s) [two-way ANOVA: *F* (2, 25) = 4.33, *p* = 0.02]. Conversely, mice that were placed on the HF1G diet spent significantly less time in the corners (156.6 ± 61.6 s) as compared to the HFD diet control group (279.3 ± 105.6 s) [two-way ANOVA: *F* (2, 25) = 3.96, *p* = 0.04)]. There was no significant difference observed between the STD (269.5 ± 149.4 s) and HF1G (203.4 ± 94.9 s) (*p* = 0.6, unpaired *t*-test) (*n* = 30). Considering potential impairments that were caused by the HFD, the total distance travelled was also taken into account. The total distances that were travelled by the STD (30.98 ± 12.09 m), HFD (26.937 ± 6.06 m), and HF1G (32.79 ± 11.8 m) groups were not statistically significant (two-way ANOVA *F* (2, 27) = 0.67, *p* = 0.51).

#### 3.2.2. Novel Object Recognition Test (NORT)

**Figure 4:**
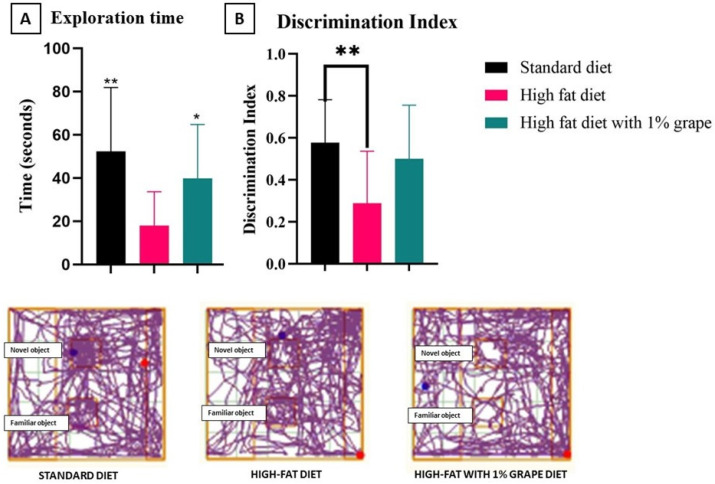
Novel Object recognition test. (**A**) Exploration time, and (**B**) Discrimination Index and track plot from the test day. Compared with the HFD group in (**A**), the amount of time that was spent exploring the novel object was higher with both the STD (*p* = 0.004) and HF1G groups (*p* = 0.02). In (**B**), the discrimination index (DI) was higher with the STD group compared with the HFD group (*p* = 0.001). When comparing the DI values, there was no statistical difference between the HF1G and STD groups (*p* = 0.46), indicating normalization. Comparing the DI values of the HFIG and HFD groups, statistical significance was not achieved (*p* = 0.07), but a trend toward normalization was apparent (Student’s *t*-test, *n* = 30) (bars represent standard deviation, * *p* < 0.05, ** *p* < 0.01). The track plots show the positioning of both the novel and familiar object in the field and the locomotor behavior of mice placed in the open field. There was no difference that was observed in distance that was traveled between the HFD and HF1G [two-way ANOVA-Dunnett’s multiple comparison *F* (2, 27) = 16, *p* = 0.86].

Compared with the HFD group (17.99 ± 15.42 s), the amount of time spent exploring the novel object (exploration time) was observed to be higher in the group of mice that were provided with the STD (52.36 ± 29.49 s) or the HF1G (39.9 6 ± 24.85 s) [two-way ANOVA *F* (2, 27) = 5, *p* = 0.02] (Figure 4). This indicates that the impaired cognitive faculties with mice that were provided with the HFD was attenuated by grape supplementation.

The discrimination index (DI) was calculated by dividing the difference of the time that was spent on the novel and familiar objects by the total time that was spent on both the objects. Based on this parameter, again, mice that were placed on the HFD (0.28 ± 0.24) were impaired relative to the mice that were placed on the STD (0.57 ± 0.20) (*p* = 0.001). There was no significant difference between the STD group and the HF1G group (0.50 ± 0.24) (*p* = 0.46), consistent with the exploration time results. Relative to the HFD group, the DI of the HF1G group did not achieve statistical significance (*p* = 0.07), but the results did support a trend of improving cognition. Considering potential impairments that are caused by the HFD, the total distance that was travelled was also taken into account. The difference between the total distance travelled by the HFD (15.05 ± 2.60 m) and the HF1G (15.95 ± 3.3 m) groups was not statistically significant [two-way ANOVA-Dunnett’s multiple comparison *F* (2, 27) = 16, *p* = 0.86]. The distance that was travelled by the STD group (25.19 ± 6.3 m) was significantly different relative to the other groups [two-way ANOVA-Dunnett’s multiple comparison *F* (2, 27) = 16, *p* = 0.00].

#### 3.2.3. Radial Arm Maze

**Figure 5:**
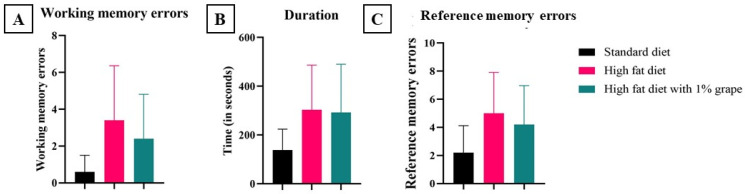
Radial Arm Maze test. (**A**) Working memory errors, (**B**) Duration of the test, and (**C**) Reference memory errors. (**A**) Working memory errors are also referred to as short-term memory errors. There were no statistical differences between the groups. (**B**) The duration that was required by the mice to complete the test (in seconds). There were no statistical differences between the high-fat diet groups and the high-fat diet that was supplemented with grape; there was a significant difference between the HFD and the STD groups (*p* = 0.01). (**C**) Reference memory errors are also referred to as long-term/spatial memory errors. There were no statistical differences between any of the groups (Student’s *t*-test assuming equal variance) (bars represent standard deviation) (Student’s *t*-test, *n* = 15).

Working memory refers to short-term memory errors. It is calculated by adding revisits to all the arms (baited or otherwise) in one session. Working memory errors that were observed in mice that were placed on the HFD (3.4 ± 3.0) trended higher than those on STD (0.6 ± 0.89), but statistical significance was not achieved due to variability (*p* = 0.07). Similarly, the HF1G group (2.4 ± 2.4) did not show a statistical difference relative to the HFD (*p* > 0.05) or the STD groups (*p* > 0.05) (Figure 5A).

The average time that each mouse takes to complete the duration test indicates intact memory. There was a significant difference that was observed between the group of mice that were provided with the STD (132.6 ± 85.6 s) versus the HFD (303.6 ± 185.2 s) (*p* < 0.05) (Figure 5B), indicating adverse effects on memory and cognition due to chronic consumption of the HFD. However, there was no improvement that was observed with mice that were provided with the HF1G (292.26 ± 198.02 s) (*p* > 0.05, Student’s *t*-test assuming equal variance).

Reference memory indicates long-term memory or spatial memory. A higher number of errors indicates impaired long-term memory. Reference memory errors that were observed in mice placed on the STD (2.2 ± 1.9), HFD (5.0 ± 2.9), HF1G (4.2 ± 12.7) showed no statistical differences (*p* > 0.05) (Figure 5C).

### 3.3. RNA-Seq Data Analysis

#### 3.3.1. Differentially Expressed Genes

**Figure 6:**
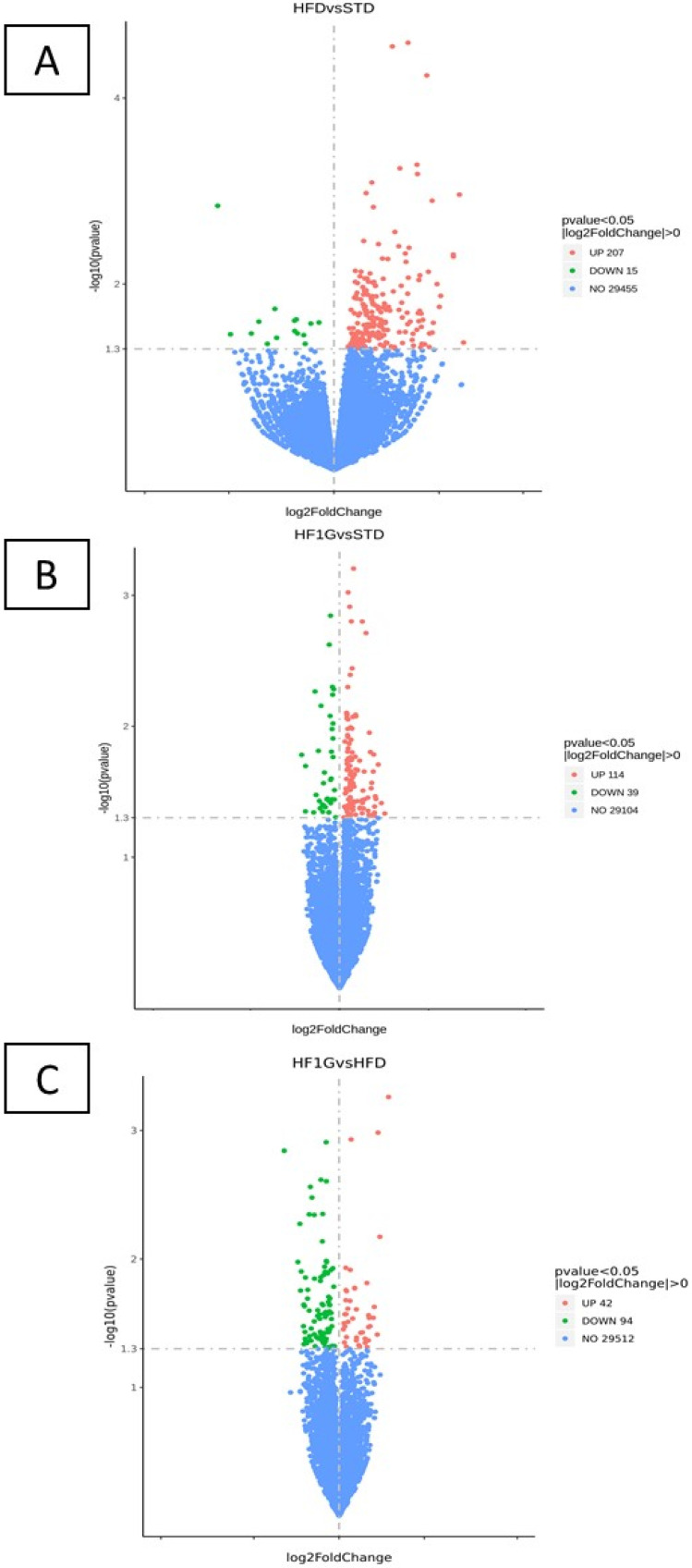
Volcano plots of gene expression across the diet groups. The dots on the chart indicate the log 2-fold change values of each gene that is differentially-expressed. The red dots indicate upregulation and the green dots indicate downregulation of these genes; blue dots indicate no statistical difference that was observed. Greater scatter along the *x*-axis shows a greater difference in the expression of the genes, as observed in the comparison between (**A**) HFD and STD (altered expression of 222 genes; 207 genes are upregulated). Less scatter along the *x*-axis was observed with (**B**) HF1G vs. STD (altered expression of 153 genes; 114 genes are upregulated) and (**C**) HF1G vs. HFD (altered expression of 136 genes; 42 genes are upregulated).

To illustrate the genetic variability, volcano plots were prepared with cut-off values of 1.3-fold change on the *x*-axis and a *p* ≤ 0.05 on the *y*-axis (Figure 6). With samples that were derived from the HFD group compared with the STD group, there were 207 genes that were upregulated and 15 genes that were downregulated. The volcano plot is more scattered over the extremes of *x*-axis which indicates a larger number of fold-changes and the magnitude of the changes (Figure 6A). However, the scatter is more tempered and of greater similarity when comparing the HF1G and STD groups (114 upregulated genes and 39 downregulated genes) (Figure 6B) and HF1G and HFD groups (42 upregulated genes and 94 downregulated genes) (Figure 6C). This indicates that the grape powder supplementation bridges the gap of the differences in genetic expression that are caused by the HFD. When comparing the STD and the HFD, there were a total of 233 genes that were differentially expressed. The identification of the up- and downregulated genes for each of these comparisons is provided in Supplement Table S1.

**Figure 7:**
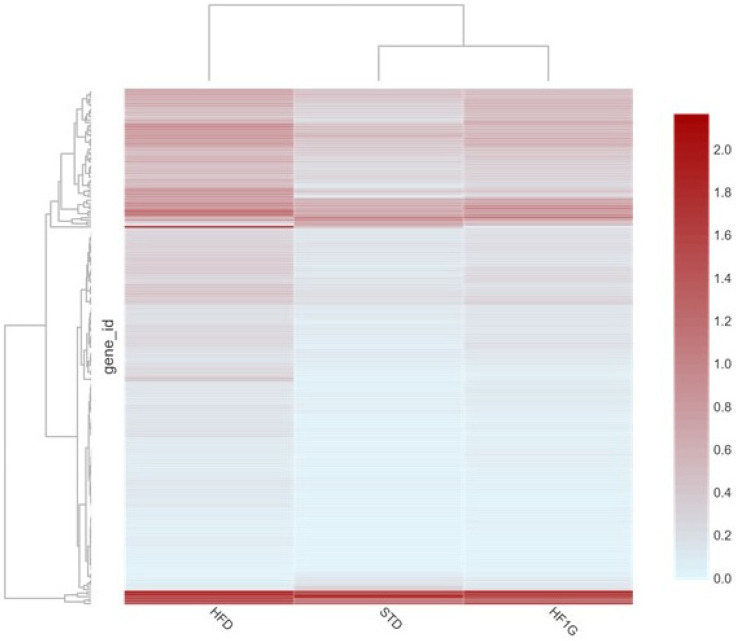
Heat map cluster analysis of the differentially expressed genes in the diet groups. The scale bar shows low to high values of log10(FPKM+1) that were obtained from the DEG list, where the red color indicates a higher expression, and the light blue color indicates a lower expression. The horizontal clustering of genes places a group of genes with similar expression under the same hierarchy. The hierarchical clustering analysis shows that the expression of genes in the brains of mice that were placed on the HFIG diet are more closely related to genes in the brains of mice that were placed on the STD and less related to genes in the brains of mice that were placed on the HFD.

To further explore these relative differences, FPKM values were normalized, and the resultant log10(FPKM+1) values were used to perform cluster analyses. As can be visually perceived from Figure 7, the panel of genes resulting from mouse brain that was derived from the HF1G group differs from the HFD group and bears a resemblance to the STD group as opposed to the HFD group. In fact, cluster analysis revealed that the expression profile of the HF1G group maps closer to the STD group than to the HFD group.

**Figure 8:**
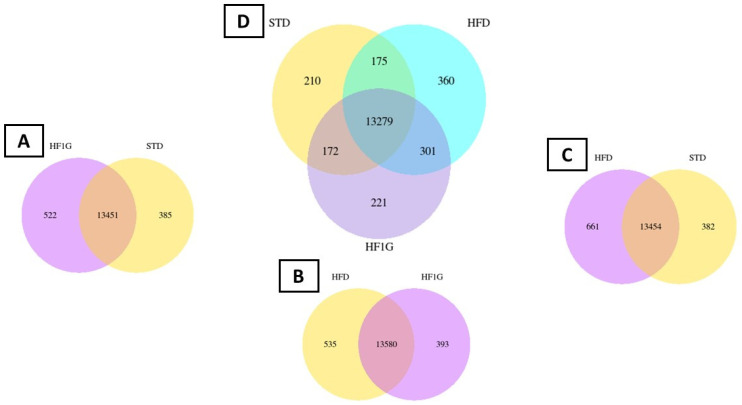
Venn diagrams of mouse brain gene expression. (**A**) Genes common between HF1G vs. STD, (**B**) Genes common between HFD vs. HF1G, and (**C**) Genes common between HFD vs. STD. (**D**) Genes common between HFD vs. STD vs. HF1G. The overlapping areas show the number of genes that were co-expressed by the respective groups, and the areas not overlapping show the number of genes that were uniquely expressed by the respective groups.

These results are further accentuated by the Venn diagrams that are shown in Figure 8. A comparison between the HF1G vs. STD showed 522 genes were uniquely expressed by HF1G and 385 genes were uniquely expressed by STD (Figure 8A). A comparison between the HFD vs. HF1G shows 535 genes were uniquely expressed by HFD and 393 genes were uniquely expressed by HF1G (Figure 8B). A comparison between the HFD vs. STD shows 661 genes were uniquely expressed by HFD and 382 genes were uniquely expressed by STD (Figure 8C). A comparison between the STD vs. HFD vs. HF1G shows 210 genes were uniquely expressed by STD, 360 genes were uniquely expressed by HFD, and 221 genes were uniquely expressed by HF1G; there were 175 genes that were similarly co-expressed between STD and HFD, 360 genes that were similarly co-expressed between HFD and HF1G, and 172 genes that were similarly co-expressed between STD and HF1G (Figure 8D).

**Figure 9:**
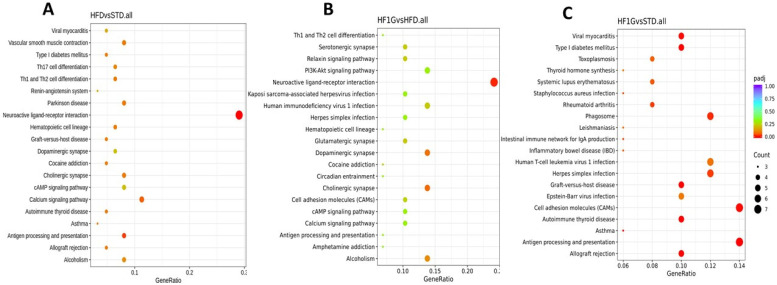
A dot graph representing the KEGG pathway analysis showing comparison between the HFD and STD (**A**), the HF1G and HFD (**B**), and the HF1G and STD (**C**) (KEGG, Kyoto Encyclopedia of Genes and Genomes).

To provide some indication of the functional relevance of these genetic alterations, enriched KEGG pathway analysis was performed comparing the HFD and STD groups (Figure 9A), the HFIG and HFG groups (Figure 9B), and the HFG1 and STD groups (Figure 9C). Greater detail of the genes contributing to this pathway analysis is provided in Supplement Table S2. Of particular note is the differentiation of “neuroactive ligand-receptor interaction” with the HFD vs. STD groups, and the HFG1 vs. HFD groups, but not the HFG1 and STD groups.

#### 3.3.2. Dopamine Receptor 2 in Whole Brain

Some specific annotations of relevance emerged when the differentially expressed gene list was processed using a gene ontology (GO) database. As summarized in Table 2, biological process regulation of dopamine secretion (GO:0014059), the molecular function of dopamine binding (GO:0035240), and the cellular component dopaminergic synapse (GO:0035240), were upregulated in the HFD group compared with the STD group, and downregulated when comparing the HFD and HF1G groups. Similarly, Panther pathway analysis demonstrated the dopamine receptor-mediated signaling pathway and the heterotrimeric G-protein signaling pathway, Gi-mediated pathways, both associated with dopamine receptor 2 activity, were upregulated within the HFD group and attenuated by grape intervention.

**Figure 10:**
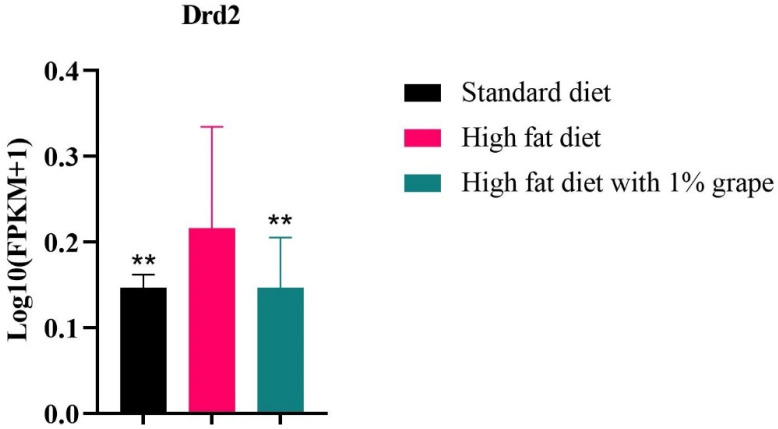
mRNA expression of Drd2 gene. Dopamine receptor 2 (Drd2) in mouse brain from the HFD (FPMK = 6.12) was significantly (*p* = 0.042) higher than mouse brain from the HF1G (FPMK = 1.75) (*p* = 0.042) or STD groups (FPMK = 1.54) (*p* = 0.00). The levels of the latter two groups (STD and HF1G) did not differ (*p* = 0.99) (Student’s *t*-test, *n* = 15) (bars represent the standard deviation, ** *p* < 0.05).

As shown in Figure 10, the mRNA expression of the Dopamine receptor 2 (Drd2) itself was significantly higher in the HFD group (FPKM value = 6.2; *p* < 0.05) than in the HF1G (FPKM value = 1.75) or the STD groups (FPKM value = 1.54). The level in the HF1G group did not differ from the STD group (*p* = 0.99).

**Figure 11:**
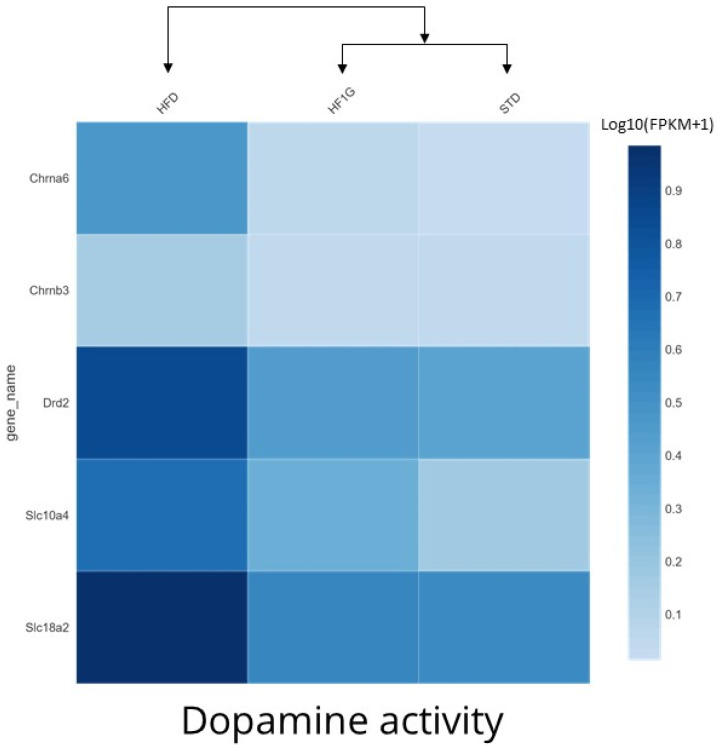
Heatmap cluster analysis of genes that are involved in the dopamine receptor 2- mediated signaling pathway in female C57BL6/J mouse brain influenced by the placement on different diets. Log10(FPKM +1) values of genes that are involved in the biological process of regulating dopamine secretion by D2 receptor, molecular function of dopamine binding, and cellular component dopamine synapse, are shown in the heatmap. As judged by cluster hierarchy, the gene expression in mouse brain that was derived from the HF1G group shows greater similarity to the STD group than the HFD group, based on the following: Dopamine receptor 2, Solute Carrier Family 18 (Vesicular Monoamine Transporter), Member 2 (SLc18a2), Opioid Receptor Kappa 1 (Oprk1), Cholinergic Receptor Nicotinic Alpha 6 Subunit (Chrna6), Cholinergic Receptor Nicotinic Beta 3 Subunit (Chrnb3), and Solute Carrier Family 10 Member 4 (SL10a4).

These relative expression levels are also reflected in the heatmap shown in Figure 11. The Drd2 signal observed with the HFD group was reduced in the HF1G group and similar to the STD group. The same pattern of upregulation that was caused by the HFD and normalization by the HF1G was observed with other genes that were involved in these annotations: Solute Carrier Family 18 (Vesicular Monoamine Transporter), Member 2 (SLc18a2), Opioid Receptor Kappa 1 (Oprk1), Cholinergic Receptor Nicotinic Alpha 6 Subunit (Chrna6), Cholinergic Receptor Nicotinic Beta 3 Subunit (Chrnb3), and Solute Carrier Family 10 Member 4 (SL10a4). Notably, taking cluster hierarchy into account, the expression of these genes in the HF1G group is similar to, and maps more closely to, those of the STD group than the HFG group.

#### 3.3.3. Genes Associated with Feeding Behavior

**Figure 12:**
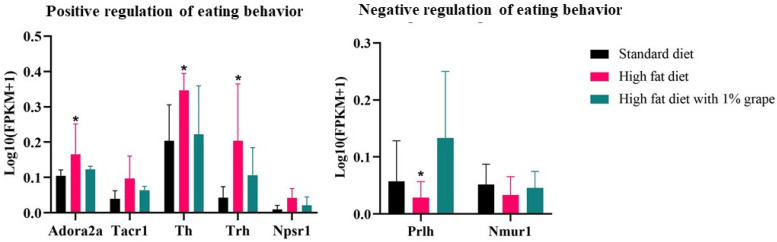
Expression of genes that are associated with the regulation of eating behaviors. Genes regulating positive feeding behaviors such as Adenosine A2a Receptor (Adora2a) (fold change = 1.57), Tachykinin Receptor 1 (Tacr1) (fold change = 1.85), Neuropeptide S Receptor 1 (Npsr1) (fold change = 2.40), Tyrosine Hydroxylase (Th) (fold change = 2.12), and Thyrotropin Releasing Hormone (Trh) (fold change = 4.42) were significantly upregulated by the HFD compared to the STD diet and normalized by downregulation upon grape intervention (HF1G). The expression of genes that negatively regulate feeding behavior, including Prolactin Releasing Hormone (Prlh) (fold change = −1.50) and Neuromedin U Receptor 1 (Nmur1) (fold change = −0.78), trended downward in the HFD group compared to the STD group. Upon grape intervention, Prlh was upregulated (fold change = 3.37) compared with the HFD group (Student’s *t*-test, *n* = 15) (the bars represent the standard deviation, * *p* <0.05, *p* <0.01).

Some genes that are associated with the positive regulation feeding behavior, such as binge eating (behaviors that mimic addictive behaviors that are associated with drug abuse), were upregulated with chronic consumption of the HFD, compared with the STD group. Upregulation that was induced by the HFD was ameliorated by the addition of grape. Examples include Adenosine A2a Receptor (Adora2a) (log2 fold change, −1.23; HF1G vs. HFD), Tachykinin Receptor 1 (Tacr1) (log2 fold change, −1.08), Tyrosine Hydroxylase (Th) (log2 fold change, −1.52), and Thyrotropin-Releasing Hormone (Trh) (log2 fold change, −2.4) (Figure 12 and Table 3).

Some genes that negatively regulate feeding behavior include Prolactin Releasing Hormone (Prlh) and Neuromedin U Receptor 1 (Nmur1). As shown in Figure 11 and summarized in Table 3, Prlh was upregulated in the HF1G group (fold change, 3.37), even higher than the STD group. Nmur1 was not significantly affected (fold change, 0.68).

## 4. Discussion

Generous dietary consumption of fruits and vegetables is broadly associated with the promotion of health benefits including a reduced risk of cognitive impairment and dementia [13]. A variety of mechanisms have been demonstrated or proposed, largely based on anti-oxidant activity and the potential of the phytochemical constitutes to modulate signaling pathways, decrease anti-inflammation, increase cerebral blood flow, etc. [14]. More recently, the potential impact of the microbiota-gut-brain axis been highlighted [15], highlighting another avenue wherein diet may influence overall health, psychological well-being, and cognitive function. Clearly, a great deal of work is still required to provide a holistic understanding of how diet and lifestyle affect health and brain function.

One of the first questions that was addressed in the current work was the selection of a model system. As described herein, we elected to employ female C57BL6/J mice. First, these mice are popularly used as an animal model for Western diet-induced obesity. They are known to be highly responsive to a HFD [16], and the progression of obesity and metabolic anomalies resembles human obesity progression [17]. Further, they are active and good at learning and completing new tasks, making them suitable for studying behavior [18]. While it is true that it would be of value to perform similar studies with male mice as well as female mice, this work was designed as a pilot to minimize number of animals. However, based on the data that were reported herein, additional studies with male mice would be of interest.

Next, direct comparisons of the variables of interest were facilitated by using a semi-synthetic standard diet as a base, which is devoid of confounding constituents that are present in commercial animal chow. Further, we elected to investigate a standardized whole grape product that is representative of what is found in the human diet, rather than studying a single phytochemical that is known to be in grapes, such as resveratrol. Since the grape product was provided in the diet, an important question is the amount to be added. We elected to supplement the high-fat diet with 1% grape powder. Although translations of dose between species is not an exact science, based on body weight, daily consumption rates, and metabolic correction factors [19], it was estimated that supplementation of the mouse diet with 1% grape powder corresponds to the daily consumption of about 60 g of fresh grapes by a human being weighing 70 kg. This quantity seems reasonable, since it is equivalent to about one-half of a normal ¾ cup serving of grapes, which is approximately 125 g.

Given this background, we set out to explore the potential of dietary grapes to prevent the cognitive decline that is associated with the chronic consumption of a high-fat diet (HFD). In addition, work was performed to explain the underlying mechanism of memory decline and behavioral changes that are caused by the consumption of a HFD, and the potential of grape supplementation to attenuate these effects. In monitoring the metabolic profile by body weight, we observed that the mice that were provided with the HFD had increased body weight compared to the STD, and the addition of grapes to the HFD did not affect the body weight progression. As described previously, the addition of grape powder in this fashion to this diet does not significantly affect the consumption rate [12]. Further, although there is some controversy regarding the potential of grape polyphenols to cross the blood-brain barrier, a recent study demonstrated that metabolites and their native chemical forms were found in brain tissue [20].

Memory decline is strongly related to aging and other neuropathophysiological conditions that are associated with aging such as Alzheimer’s disease and dementia [21]. Studies supporting the protective effects of grape interventions on memory decline have been reported [21,22,23]. In our work, we used a radial arm maze to explore the impact of the HFD and the HF1G on working memory (short-term memory), intact memory, and spatial memory (long-term memory), relative to STD. No significant differences between any of the groups was observed with the spatial memory test, although the HFD group trended toward a higher number of errors. Similarly, there was a strong trend of increased working memory errors with the HFD group, but statistical significance was not achieved (*p* = 0.07). The HF1G did not differ from the HFD group. For intact memory, the HFD group showed impairment relative to the STD group, but improvement was not observed in the HF1G group. These data strongly suggest memory decline due to consumption of a HFD, but only weakly suggest any improvement due to the addition of grape to the diet.

In a preliminary manner, we surveyed the relative level of gene expression that is associated with long-term potentiation (LTP) given this is widely accepted as a cellular mechanism underlying memory processes. However, differences between the STD, HFD, and HF1G groups were not identified when a cadre of LTP-related genes that were reported in the literature [24,25,26] were investigated (data not shown). Nonetheless, this work may bear repeating with a more highly powered study design and more refined analysis of temporal sequence.

Beyond memory deficits and enhancements, we were interested in the test results that correlate with emotionality. It is known that diet plays a vital role in mental health; continued consumption of a HFD affects the emotionality of subject animals. Mice that were placed on a HFD for six months show depression and anxiety-like behavior [27,28]. We conducted an open field test to study this behavior. Based on our observations, it is clear that the mice that were provided with the HFD linger in the corners of the field, unlike the mice that were provided with the STD diet. The track plot demonstrates that the mice that were provided with the STD explore throughout the field, while the mice that were placed on the HFD have minimal explorative tendencies. Notably, the behavioral impairments that were observed with the HFD group were significantly attenuated by grape supplementation. These results are consistent with studies that were conducted with rats wherein grape powder treatments improved several behavioral aberrations such as post-traumatic disorders, anxiety-like behavior, and depression [6,29,30].

The potential of dietary grapes to attenuate impaired cognitive faculties with mice that were provided with the HFD was also demonstrated with the novel object recognition test (NORT). Here, the amount of time spent exploring a novel object with mice that were provided with the STD or the HF1G exceeded that of the HFD group. Similarly, considering the discrimination index (DI) (the difference in time spent on the novel and familiar objects divided by the total time spent on both the objects), the HFD showed significant impairment relative to the STD group. However, improvement was realized when grape was added to the HFD, since there was no significant difference between the STD and HF1G groups.

Thus, these behavior tests suggest cognitive deficits that result from the provision of a HFD, and there are at least subtle indications that the addition of grape to a HFD can help to ameliorate these deficits. Naturally, it is of great interest to know the underlying mechanisms by which diet may affect cognition, behavior, etc. We elected to employ global gene expression to explore the brain patterns with the groups of mice that had been placed on the STD, HFD, and HF1G.

First, it may be noted that we elected to evaluate a whole mouse brain based on the premise of simultaneous analysis of total brain response and status was of greatest interest at this phase of investigation. Of course, the mouse brain can be divided into many regions, and analysis of region-specific alterations may be explored in future work. However, considering that changes in one region of the brain could lead to changes in other regions of the brain, we consider global analysis of value in the context of illustrating a global change which, in total, may lead to response.

Following this approach, the differential patterns that were observed with these groups of mice were remarkable. First of all, the expression profile comparisons of the individual groups (e.g., STD vs. HFD; STD vs. HF1G; and HFD vs. HF1G) all showed major divergence. Even moreover, a comparison of all three groups revealed unique alteration of gene expression, corresponding to 210, 360, and 221 differences in the STD, HFD, and HF1G groups, respectively (Figure 8, Supplement Table S1).

Additionally, and perhaps more importantly, as indicated by cluster analysis and volcano plots, the gene expression pattern that was observed with the HF1G group mapped more closely with the STD group than with the HFD group. These data are consistent with some behavioral responses in which mice that were given the HF1G diet more closely mimicked the STD group and may correlate with an underlying mechanism.

With datasets of this type, a great many factors can be analyzed. However, one potentially important feature that was shown by our RNA-Seq data relates to dopamine-related genes. D2 receptors are inhibitory, they inhibit adenyl cyclase and calcium channels, and decrease the dopamine release in neuronal synapses. Dopamine activity dysregulation is linked to behavioral disturbances such as depression and anxiety [31]. In a study that was reported by Cagniard et al. [32] with mice, it was found that a chronic increase of dopamine levels correlates with increased binge eating behavior and limited learning abilities. In addition, mice with increased dopamine receptor 2 activity exhibited reduced exploratory behavior and impaired locomotor activity [33,34].

In our study, the mRNA expression of genes such as Dopamine receptor 2 (Drd2), Tyrosine Hydroxylase (Th), Adenosine A2a Receptor (Adora), and Opioid Receptor Kappa 1 (Oprk1) that are responsible for dopamine activity, were dysregulated by the HFD, and intervention by the addition of grapes to the diet normalized this dysregulation.

Adora gene expression also plays an essential role in dopamine activity, serotonin release in the hippocampal region, and has several effects on adenylyl cyclase activity. The dysregulation of Adora2a causes serious neurological issues that presented as cognitive dysfunction, motor disabilities, and self-injurious behavior [35]. Here we show that the dysregulation of Adora in the HFD group was attenuated by grape intervention.

KEGG pathway analyses provide additional support for the normalization of pathways that are dysregulated by consumption of the HFD. For example, the KEGG pathway indicative of alcoholism (KEGG ID mmu05310) was altered by the HFD compared to the STD. In the HFD group, five genes (Th/Gng4/Slc18a2/Drd2/Adora2a) were upregulated, but expression was normalized by grape supplementation (*p* = 0.01). Similarly, genes that are suggestive of cocaine addiction (KEGG ID: mmu05030) were upregulated in the HFD group compared with the STD group, but the expression of genes that are involved in these pathways (Th/Slc18a2/Drd2) were downregulated (or tempered) in the HF1G group (*p* = 0.01). As another example, dopaminergic synapse (KEGG ID: mmu04728) was altered due to the HFD compared with the STD, but the expression of genes that are involved in these pathways (Th/Gng4/Slc18a2/Drd2) were downregulated (or tempered) by HF1G (*p* = 0.002). Perhaps most notable, neuroactive ligand-receptor interaction (KEGG ID: mmu04080) was altered due to the HFD compared to the STD, in that with the HFD group, 18 out of 62 genes in the signaling pathway (Chrna6/Htr2c/Chrm2/Drd2/Grm8/Sstr5/Tacr1/Avpr1a/Oprk1/Chrna10/.

Chrnb3/Hcrtr1/Adora2a/Brs3/Glra3/Gh/Chrm5/Tacr3) were upregulated. The expression of seven genes (Gh/Chrna6/Chrm2/Grm8/Rxfp2/Drd2/Trhr2) that are involved in these pathways were normalized by grape supplementation (*p* = 0.00); there was no significant difference between the STD and HF1G groups.

Of course, these data regarding the expression analysis are presumptive in nature, and more detailed investigations are required in the future. For example, the information that is provided above points to the importance of directly analyzing the level of dopamine and related species using methods such as HPLC.

Other genes, such as Tachykinin Receptor 1 (Tacr1), Neuropeptide S Receptor 1 (Npsr1), Tyrosine Hydroxylase (Th), and Thyrotropin Releasing Hormone (Trh), that regulate positive feeding behaviors, were upregulated in mice that were fed the HFD compared to the STD. These factors can also disturb circadian rhythm.

With various degrees of efficacy, grape supplementation altered the HFD-induced dysregulation of this family of genes (Table 3). Of potential relevance, in some feeding studies that were conducted with mice, the total amount of food that was consumed was not altered, but a large amount of food was consumed altogether in a small frame of time [36,37]. We did not evaluate such features in detail, but it is clear that grape supplementation did not influence the body weight, although it did alter some genes that are associated with the control of such feeding behaviors.

In sum, our findings suggest that grape supplementation alters gene expression and has beneficial effects on anxiety-like behavior and feeding behavior that contribute to better cognition and memory in mice that are exposed to a high-fat diet for a long duration. How these results correlate with human consumption of grapes remains to be seen. Of note, however, randomized clinical trials that were conducted with healthy human subjects across all demographic age groups demonstrated that consumption of grapes augmented brain function and cognition [38,39]. Also, in a double-blind study that was conducted with subjects 68–76 years of age with mild cognitive decline, it was observed that grape intervention played a role in preventing the metabolic deterioration of early pathological conditions of Alzheimer’s disease [40]. Perhaps some of the results that are reported herein can eventually help to provide a biomolecular basis for such responses.

Finally, the cadre of mechanisms that are normally taken into account when considering the health benefits of enhanced fruit and vegetable consumption are generally related to the direct action of phytochemical constituents. Here, we emphasize the astounding ability of diet to modulate the actual phenotypic expression in brain and other tissues. This mode of action requires serious deliberation. The proverbial saying ‘You are what you eat’, originally attributed to the French lawyer Anthelme Brillat-Savarin (ca. 1826), was likely inspired by the nutritive value of food, as well as the metabolic conversion of food into human cellular and body parts. The notion of diet leading to phenotypic changes, which, in turn, may induce cognitive alterations, adds another dimension and even more profound significance to the axiom ‘You are what you eat’.

## Figures and Tables

**Table 1:** Diet constituents.

	Standard Diet	High-Fat Diet	High-Fat Diet with 1% Grape Powder
	STD	HFD	HF1G
	g/kg Diet		
Casein	195	195	191
DL-Methionine	3	3	3
Sucrose	191	191	191
Dextrose (anhydrous)	66	65	62.454
Fructose	66	65	62.454
Corn Starch	235	167.43	166.22
Anhydrous Milkfat	30	210	210
Cholesterol	0	1.5	1.5
Cellulose	50	50	50
Mineral Mix, AIN-76 (170915)	35	35	35
Calcium Carbonate	4	4	4
Potassium Citrate (monohydrate)	4	4	4
Vitamin Mix, Teklad (40060)	10	10	10
Ethoxyquin (antioxidant)	0.04	0.04	0.04
Grape Powder, freeze-dried	0	0	10

**Table 2:** Comparison of dopamine signaling genes among groups [biological process regulation of dopamine secretion (GO:0014059); molecular function of dopamine binding (GO:0035240); cellular component dopaminergic synapse (GO:0035240); Panther pathways related to dopamine receptor 2-mediated signaling pathways which inhibit dopamine release (P05912; P00026)].

GO ID	Annotation		HFD vs. STD	HF1G vs. HFD
GO:0014059	Biological Process	Regulation of dopamine secretion by D2 receptor	Upregulated	Downregulated
GO:0098691	Cellular Component	Dopaminergic synapse	Upregulated	Downregulated
GO:0035240	Molecular Function	Dopamine binding	Upregulated	Downregulated
P05912	Panther pathways	Dopamine receptor-mediated signaling pathway	Upregulated	Downregulated
P00026	Panther Pathways	Heterotrimeric G-protein signaling pathway-Gi alpha and Gs alpha mediated pathway	Upregulated	Downregulated

**Table 3:** Eating behaviors.

GO Annotation	Gene Name	HFD vs. STD		HF1G vs. HFD	
		Fold Change	*p*-Value	Fold Change	*p*-Value
Positive feeding behavior	Trh	4.42	0.00	−2.43	0.03
	Th	2.12	0.00	−1.52	0.07
	Adora2a	1.57	0.02	−1.23	0.08
	Tacr1	1.85	0.01	−1.07	0.12
	Npsr1	2.40	0.03	−0.34	0.3
Negative feeding behavior	Prlh	−1.50	0.48	3.37	0.04
	Nmur1	−0.71	0.53	0.68	0.7

## Data Availability

Data is contained within the article and supplementary material.

## Supplementary Materials

The following supporting information can be downloaded at: https://www.mdpi.com/article/10.3390/antiox11020414/s1, Table S1: Differentially Expressed Gene List; Table S2: KEGG Pathway Analysis.

## References

[1] Shook L.L., Kislal S., Edlow A.G. (2020). Fetal brain and placental programming in maternal obesity: A review of human and animal model studies. Prenat. Diagn..

[2] Tantot F., Parkes S.L., Marchand A.R., Boitard C., Naneix F., Layé S., Trifilieff P., Coutureau E., Ferreira G. (2017). The effect of high-fat diet consumption on appetitive instrumental behavior in rats. Appetite.

[3] Pezzuto J.M. (2011). The phenomenon of resveratrol: Redefining the virtues of promiscuity. Ann. N. Y. Acad. Sci..

[4] Bulloj A., Finnemann S.C., Pezzuto J.M. (2016). Grapes and Vision. Grapes and Health.

[5] Xia E.-Q., Deng G.-F., Guo Y.-J., Li H.-B. (2010). Biological activities of polyphenols from grapes. Int. J. Mol. Sci..

[6] Patki G., Ali Q., Pokkunuri I., Asghar M., Salim S. (2015). Grape powder treatment prevents anxiety-like behavior in a rat model of aging. Nutr. Res..

[7] Calapai G., Bonina F., Bonina A., Rizza L., Mannucci C., Arcoraci V., Laganà G., Alibrandi A., Pollicino C., Inferrera S. (2017). A Randomized, Double-Blinded, Clinical Trial on Effects of a Vitis vinifera Extract on Cognitive Function in Healthy Older Adults. Front. Pharmacol..

[8] Pezzuto J.M. (2008). Grapes and human health: A perspective. J. Agric. Food Chem..

[9] Maher P., Pezzuto J.M. (2016). Grapes and the Brain. Grapes and Health.

[10] van Breemen R.B., Wright B., Li Y., Nosal D., Burton T., Pezzuto J.M. (2016). Standardized Grape Powder for Basic and Clinical Research. Grapes and Health.

[11] Meziane H., Ouagazzal A.M., Aubert L., Wietrzych M., Krezel W. (2007). Estrous cycle effects on behavior of C57BL/6J and BALB/cByJ female mice: Implications for phenotyping strategies. Genes Brain Behav..

[12] Joshi T., Patel I., Kumar A., Donovan V., Levenson A.S. (2020). Grape Powder Supplementation Attenuates Prostate Neoplasia Associated with Pten Haploinsufficiency in Mice Fed High-Fat Diet. Mol. Nutr. Food Res..

[13] Jiang X., Huang J., Song D., Deng R., Wei J., Zhang Z. (2017). Increased Consumption of Fruit and Vegetables Is Related to a Reduced Risk of Cognitive Impairment and Dementia: Meta-Analysis. Front. Aging Neurosci..

[14] Carrillo J.Á., Zafrilla M.P., Marhuenda J. (2019). Cognitive Function and Consumption of Fruit and Vegetable Polyphenols in a Young Population: Is There a Relationship?. Foods.

[15] Toribio-Mateas M. (2018). Harnessing the Power of Microbiome Assessment Tools as Part of Neuroprotective Nutrition and Lifestyle Medicine Interventions. Microorganisms.

[16] Wang C.-Y., Liao J.K. (2012). A mouse model of diet-induced obesity and insulin resistance. Methods Mol. Biol..

[17] Siersbæk M.S., Ditzel N., Hejbøl E.K., Præstholm S.M., Markussen L.K., Avolio F., Li L., Lehtonen L., Hansen A.K., Schrøder H.D. (2020). C57BL/6J substrain differences in response to high-fat diet intervention. Sci. Rep..

[18] Bryant C.D. (2011). The blessings and curses of C57BL/6 substrains in mouse genetic studies. Ann. N. Y. Acad. Sci..

[19] Nair A.B., Jacob S. (2016). A simple practice guide for dose conversion between animals and human. J. Basic Clin. Pharm..

[20] Bensalem J., Dudonné S., Gaudout D., Servant L., Calon F., Desjardins Y., Layé S., Lafenetre P., Pallet V. (2018). Polyphenol-rich extract from grape and blueberry attenuates cognitive decline and improves neuronal function in aged mice. J. Nutr. Sci..

[21] Albert M.S. (2002). Memory decline: The boundary between aging and age-related disease. Ann. Neurol..

[22] Berahmand F., Anoush G., Hosseini M.J., Anoush M. (2020). Grape Seed Oil as a Natural Therapy in Male Rats with Alzheimer’s Diseases. Adv. Pharm. Bull..

[23] Lin B., Hasegawa Y., Takane K., Koibuchi N., Cao C., Kim-Mitsuyama S. (2016). High-Fat-Diet Intake Enhances Cerebral Amyloid Angiopathy and Cognitive Impairment in a Mouse Model of Alzheimer’s Disease, Independently of Metabolic Disorders. J. Am. Heart Assoc..

[24] Ryan M.M., Ryan B., Kyrke-Smith M., Logan B., Tate W.P., Abraham W.C., Williams J.M. (2012). Temporal profiling of gene networks associated with the late phase of long-term potentiation in vivo. PLoS ONE.

[25] Dudek S.M., Fields R.D. (1999). Gene Expression in Hippocampal Long-Term Potentiation. Neuroscientist.

[26] Lisachev P.D., Shtark M.B., Stuchlik A. (2018). Long-Term Potentiation-Associated Gene Expression: Involvement of the Tumour Protein p53. The Hippocampus: Plasticity and Functions.

[27] Haleem D.J., Mahmood K. (2021). Brain serotonin in high-fat diet-induced weight gain, anxiety and spatial memory in rats. Nutr. Neurosci..

[28] Krishna S., Keralapurath M.M., Lin Z., Wagner J.J., de La Serre C.B., Harn D.A., Filipov N.M. (2015). Neurochemical and electrophysiological deficits in the ventral hippocampus and selective behavioral alterations caused by high-fat diet in female C57BL/6 mice. Neuroscience.

[29] Solanki N., Alkadhi I., Atrooz F., Patki G., Salim S. (2015). Grape powder prevents cognitive, behavioral, and biochemical impairments in a rat model of posttraumatic stress disorder. Nutr. Res..

[30] Allam F., Dao A.T., Chugh G., Bohat R., Jafri F., Patki G., Mowrey C., Asghar M., Alkadhi K.A., Salim S. (2013). Grape powder supplementation prevents oxidative stress-induced anxiety-like behavior, memory impairment, and high blood pressure in rats. J. Nutr..

[31] Ford C.P. (2014). The role of D2-autoreceptors in regulating dopamine neuron activity and transmission. Neuroscience.

[32] Cagniard B., Balsam P.D., Brunner D., Zhuang X. (2006). Mice with Chronically Elevated Dopamine Exhibit Enhanced Motivation, but not Learning, for a Food Reward. Neuropsychopharmacology.

[33] Phillips T.J., Brown K.J., Burkhart-Kasch S., Wenger C.D., Kelly M.A., Rubinstein M., Grandy D.K., Low M.J. (1998). Alcohol preference and sensitivity are markedly reduced in mice lacking dopamine D2 receptors. Nat. Neurosci..

[34] Han J., Nepal P., Odelade A., Freely F.D., Belton D.M., Graves J.L., Maldonado-Devincci A.M. (2021). High-Fat Diet-Induced Weight Gain, Behavioral Deficits, and Dopamine Changes in Young C57BL/6J Mice. Front. Nutr..

[35] Bertelli M., Cecchin S., Lapucci C., Jacomelli G., Jinnah H.A., Pandolfo M., Micheli V. (2006). Study of the adenosinergic system in the brain of HPRT knockout mouse (Lesch–Nyhan disease). Clin. Chim. Acta.

[36] Pankevich D.E., Teegarden S.L., Hedin A.D., Jensen C.L., Bale T.L. (2010). Caloric Restriction Experience Reprograms Stress and Orexigenic Pathways and Promotes Binge Eating. J. Neurosci..

[37] Jang H., Lee G., Kong J., Choi G., Park Y.J., Kim J.B. (2012). Feeding Period Restriction Alters the Expression of Peripheral Circadian Rhythm Genes without Changing Body Weight in Mice. PLoS ONE.

[38] Haskell-Ramsay C.F., Stuart R.C., Okello E.J., Watson A.W. (2017). Cognitive and mood improvements following acute supplementation with purple grape juice in healthy young adults. Eur. J. Nutr..

[39] Bird R.J., Hoggard N., Aceves-Martins M. (2021). The effect of grape interventions on cognitive and mental performance in healthy participants and those with mild cognitive impairment: A systematic review of randomized controlled trials. Nutr. Rev..

[40] Lee J., Torosyan N., Silverman D.H. (2017). Examining the impact of grape consumption on brain metabolism and cognitive function in patients with mild decline in cognition: A double-blinded placebo controlled pilot study. Exp. Gerontol..

