# Supplementary material for: Effect of Dietary Grapes on Female C57BL6/J Mice Consuming a High-Fat Diet: Behavioral and Genetic Changes
Source: Antioxidants (Basel). 2022 Feb 18;11(2):414. doi: 10.3390/antiox11020414 (PMC8868599; doi:10.3390/antiox11020414)
Supplement: Supplementary file 1 [file antioxidants-11-00414-s001.zip › antioxidants-1558109-SM/S1-Differentially Expressed Gene list.pdf]

S1: Differentially Expressed Genes List
(Comparison between HFD vs STD, HF1GvsHFD, HF1GvsSTD)

HFD vs STD							
gene_id	gene_name	HFD_fpkm	STD_fpkm	log2FoldCh	pvalue	gene_bioty	gene_description
ENSMUSG00000099342	Gm18180	26.02039	2.219134	3.52659	2.54E-05	processed_	predicted gene, 18180 [Source:MGI Symbol;Acc:MGI:5010365]
ENSMUSG00000110086	Gm45623	69.4047	10.11226	2.779378	2.78E-05	protein_co	predicted gene 45623 [Source:MGI Symbol;Acc:MGI:5791459]
ENSMUSG00000005892	Trh	164.2203	7.63788	4.420478	5.70E-05	protein_co	thyrotropin releasing hormone [Source:MGI Symbol;Acc:MGI:9882
ENSMUSG00000053441	Adamts19	69.94576	4.512242	3.949256	0.000522	protein_co	a disintegrin-like and metallopeptidase (reprolysin type) with thror
ENSMUSG00000035238	Kcnk15	31.32811	3.527102	3.137269	0.000571	protein_co	potassium channel, subfamily K, member 15 [Source:MGI Symbol;A
ENSMUSG00000079845	Xlr4a	25.17773	1.573481	3.974585	0.000657	protein_co	X-linked lymphocyte-regulated 4A [Source:MGI Symbol;Acc:MGI:35
ENSMUSG00000047606	Ankrd34c	147.019	42.20922	1.803564	0.00081	protein_co	ankyrin repeat domain 34C [Source:MGI Symbol;Acc:MGI:2685617
ENSMUSG00000032744	Heyl	161.1945	55.80153	1.531396	0.001053	protein_co	hairy/enhancer-of-split related with YRPW motif-like [Source:MGI S
ENSMUSG00000086096	Gm12688	22.05788	0.368144	5.970172	0.001093	lincRNA	predicted gene 12688 [Source:MGI Symbol;Acc:MGI:3650434]
ENSMUSG00000028736	Pax7	49.00228	1.912967	4.672277	0.001272	protein_co	paired box 7 [Source:MGI Symbol;Acc:MGI:97491]
ENSMUSG00000110469	Gm10358	0	8.479736	-5.53183	0.001443	protein_co	predicted gene 10358 [Source:MGI Symbol;Acc:MGI:3708786]
ENSMUSG00000050473	Slc35d3	76.81208	20.90349	1.877153	0.001488	protein_co	solute carrier family 35, member D3 [Source:MGI Symbol;Acc:MGI:
ENSMUSG00000054667	Irs4	65.56378	8.851331	2.899888	0.002756	protein_co	insulin receptor substrate 4 [Source:MGI Symbol;Acc:MGI:1338009
ENSMUSG00000030307	Slc6a11	4731.989	1772.459	1.41678	0.00345	protein_co	solute carrier family 6 (neurotransmitter transporter, GABA), mem
ENSMUSG00000000214	Th	788.7003	181.5853	2.119087	0.003728	protein_co	tyrosine hydroxylase [Source:MGI Symbol;Acc:MGI:98735]
ENSMUSG00000029219	Slc10a4	149.0776	17.60469	3.08631	0.003928	protein_co	solute carrier family 10 (sodium/bile acid cotransporter family), me
ENSMUSG00000056025	Clca3a1	32.02355	2.718566	3.571515	0.004072	protein_co	chloride channel accessory 3A1 [Source:MGI Symbol;Acc:MGI:1316
ENSMUSG00000014030	Pax5	35.44072	3.414199	3.395416	0.004701	protein_co	paired box 5 [Source:MGI Symbol;Acc:MGI:97489]
ENSMUSG00000019787	Trdn	18.01783	0.322827	5.676668	0.004832	protein_co	triadin [Source:MGI Symbol;Acc:MGI:1924007]
ENSMUSG00000031491	Chrna6	105.6233	2.072912	5.683638	0.00507	protein_co	cholinergic receptor, nicotinic, alpha polypeptide 6 [Source:MGI Sy
ENSMUSG00000009734	Pou6f2	30.30753	6.102383	2.307822	0.005324	protein_co	POU domain, class 6, transcription factor 2 [Source:MGI Symbol;Ac
ENSMUSG00000087075	Lbhd2	107.7959	18.21452	2.562743	0.005384	protein_co	LBH domain containing 2 [Source:MGI Symbol;Acc:MGI:2685744]
ENSMUSG00000036480	Prss56	36.36401	3.409536	3.427112	0.00578	protein_co	protease, serine 56 [Source:MGI Symbol;Acc:MGI:1916703]
ENSMUSG00000038370	Pcp4l1	3041.849	1504.322	1.015805	0.007224	protein_co	Purkinje cell protein 4-like 1 [Source:MGI Symbol;Acc:MGI:1913675
ENSMUSG00000079330	Lemd1	175.7175	72.1333	1.286956	0.007373	protein_co	LEM domain containing 1 [Source:MGI Symbol;Acc:MGI:1922403]
ENSMUSG00000045518	Onecut3	7.910973	0.368144	4.494076	0.007393	protein_co	one cut domain, family member 3 [Source:MGI Symbol;Acc:MGI:18
ENSMUSG00000045534	Kcna5	87.93619	26.805	1.714229	0.007709	protein_co	potassium voltage-gated channel, shaker-related subfamily, memb
ENSMUSG00000001496	Nkx2-1	26.87028	1.556821	4.088835	0.008079	protein_co	NK2 homeobox 1 [Source:MGI Symbol;Acc:MGI:108067]
ENSMUSG00000030098	Grip2	869.3617	368.3439	1.239144	0.008278	protein_co	glutamate receptor interacting protein 2 [Source:MGI Symbol;Acc:I

ENSMUSG00000114442	F630042J09R	11.399	0.985141	3.538197	0.008575	lincRNA	RIKEN cDNA F630042J09 gene [Source:MGI Symbol;Acc:MGI:36421
ENSMUSG00000104674	Gm42756	111.6055	40.32585	1.471078	0.008729	antisense	predicted gene 42756 [Source:MGI Symbol;Acc:MGI:5662893]
ENSMUSG00000055471	Alk	160.5807	54.01225	1.573243	0.00873	protein_co	anaplastic lymphoma kinase [Source:MGI Symbol;Acc:MGI:103305
ENSMUSG00000091705	H2-Q2	38.3201	7.570295	2.345097	0.008791	protein_co	histocompatibility 2, Q region locus 2 [Source:MGI Symbol;Acc:MG
ENSMUSG00000073804	Nps	10.95556	0.690971	4.042177	0.009062	protein_co	neuropeptide S [Source:MGI Symbol;Acc:MGI:3642232]
ENSMUSG00000027210	Meis2	1579.349	507.7927	1.636994	0.00944	protein_co	Meis homeobox 2 [Source:MGI Symbol;Acc:MGI:108564]
ENSMUSG00000024610	Cd74	192.3594	95.36365	1.013553	0.009499	protein_co	CD74 antigen (invariant polypeptide of major histocompatibility co
ENSMUSG00000018822	Sfrp5	196.2755	52.47823	1.903935	0.009518	protein_co	secreted frizzled-related sequence protein 5 [Source:MGI Symbol;A
ENSMUSG00000060284	Sp7	120.4749	33.7659	1.833096	0.009619	protein_co	Sp7 transcription factor 7 [Source:MGI Symbol;Acc:MGI:2153568]
ENSMUSG00000057969	Sema3b	220.2039	101.4021	1.121468	0.009781	protein_co	sema domain, immunoglobulin domain (Ig), short basic domain, se
ENSMUSG00000092528	Nlrp1c-ps	5.411148	0	4.903701	0.010024	transcribe	NLR family, pyrin domain containing 1C, pseudogene [Source:MGI S
ENSMUSG00000040856	Dlk1	674.5882	170.9219	1.981312	0.010443	protein_co	delta like non-canonical Notch ligand 1 [Source:MGI Symbol;Acc:M
ENSMUSG00000026308	Klhl30	31.52079	3.471513	3.200305	0.010458	protein_co	kelch-like 30 [Source:MGI Symbol;Acc:MGI:1918038]
ENSMUSG00000021303	Gng4	1274.843	325.3696	1.969963	0.010484	protein_co	guanine nucleotide binding protein (G protein), gamma 4 [Source:N
ENSMUSG00000090698	Apold1	127.5426	49.07404	1.375791	0.010799	protein_co	apolipoprotein L domain containing 1 [Source:MGI Symbol;Acc:MG
ENSMUSG00000054556	Gm4876	51.25612	17.64751	1.533504	0.01171	antisense	predicted gene 4876 [Source:MGI Symbol;Acc:MGI:3647654]
ENSMUSG00000041380	Htr2c	971.5277	356.2678	1.447671	0.011791	protein_co	5-hydroxytryptamine (serotonin) receptor 2C [Source:MGI Symbol;
ENSMUSG00000063935	Zar1	20.12013	2.790814	2.824535	0.012029	protein_co	zygote arrest 1 [Source:MGI Symbol;Acc:MGI:2180337]
ENSMUSG00000036594	H2-Aa	74.89168	35.14224	1.095331	0.012459	protein_co	histocompatibility 2, class II antigen A, alpha [Source:MGI Symbol;A
ENSMUSG00000107089	Gm43443	11.5804	1.059114	3.507348	0.012732	TEC	predicted gene 43443 [Source:MGI Symbol;Acc:MGI:5663580]
ENSMUSG00000062393	Dgkk	119.1622	23.91528	2.319705	0.012926	protein_co	diacylglycerol kinase kappa [Source:MGI Symbol;Acc:MGI:3580254
ENSMUSG00000045613	Chrm2	313.9887	118.3145	1.409613	0.01299	protein_co	cholinergic receptor, muscarinic 2, cardiac [Source:MGI Symbol;Acc
ENSMUSG00000086794	Gm11642	30.20749	9.954037	1.605893	0.013041	processed_	predicted gene 11642 [Source:MGI Symbol;Acc:MGI:3651889]
ENSMUSG00000081849	Gm15847	6.092234	0	5.081693	0.013418	transcribe	predicted gene 15847 [Source:MGI Symbol;Acc:MGI:3783237]
ENSMUSG00000025094	Slc18a2	619.9745	174.1795	1.832137	0.013514	protein_co	solute carrier family 18 (vesicular monoamine), member 2 [Source:
ENSMUSG00000036718	Micall2	73.54502	35.90373	1.034047	0.013525	protein_co	MICAL-like 2 [Source:MGI Symbol;Acc:MGI:2444818]
ENSMUSG00000032259	Drd2	297.16	73.76893	2.010815	0.013579	protein_co	dopamine receptor D2 [Source:MGI Symbol;Acc:MGI:94924]
ENSMUSG00000024376	Epb41l4a	146.4682	74.53214	0.97578	0.014084	protein_co	erythrocyte membrane protein band 4.1 like 4a [Source:MGI Symb
ENSMUSG00000103477	5930409G06I	58.96989	17.27082	1.774552	0.014233	TEC	RIKEN cDNA 5930409G06 gene [Source:MGI Symbol;Acc:MGI:1924
ENSMUSG00000024211	Grm8	213.7912	101.106	1.081189	0.014345	protein_co	glutamate receptor, metabotropic 8 [Source:MGI Symbol;Acc:MGI:
ENSMUSG00000037568	Vash2	91.43219	37.79322	1.277376	0.014674	protein_co	vasohibin 2 [Source:MGI Symbol;Acc:MGI:2444826]
ENSMUSG00000038112	AW551984	356.9414	102.6192	1.799126	0.014772	protein_co	expressed sequence AW551984 [Source:MGI Symbol;Acc:MGI:214
ENSMUSG00000031285	Dcx	606.4526	170.582	1.829777	0.015773	protein_co	doublecortin [Source:MGI Symbol;Acc:MGI:1277171]
ENSMUSG00000050824	Sstr5	12.03474	1.279311	3.224825	0.0159	protein_co	somatostatin receptor 5 [Source:MGI Symbol;Acc:MGI:894282]

ENSMUSG00000082361	Btc	15.40989	2.893445	2.403101	0.016243	protein_co	betacellulin, epidermal growth factor family member [Source:MGI
ENSMUSG00000068196	Col8a1	148.5076	57.06176	1.382462	0.016473	protein_co	collagen, type VIII, alpha 1 [Source:MGI Symbol;Acc:MGI:88463]
ENSMUSG00000030043	Tacr1	114.3468	31.72294	1.854985	0.016504	protein_co	tachykinin receptor 1 [Source:MGI Symbol;Acc:MGI:98475]
ENSMUSG00000038805	Six3	143.0294	36.65867	1.964783	0.016975	protein_co	sine oculis-related homeobox 3 [Source:MGI Symbol;Acc:MGI:1027
ENSMUSG00000029032	Arhgef16	40.36573	14.51798	1.484578	0.017393	protein_co	Rho guanine nucleotide exchange factor (GEF) 16 [Source:MGI Sym
ENSMUSG00000094152	Slc6a16	40.52046	9.466602	2.100009	0.017589	protein_co	solute carrier family 6, member 16 [Source:MGI Symbol;Acc:MGI:2
ENSMUSG00000020123	Avpr1a	13.5391	2.219134	2.593355	0.017613	protein_co	arginine vasopressin receptor 1A [Source:MGI Symbol;Acc:MGI:18
ENSMUSG00000051980	Casr	5.813155	0	5.013069	0.017628	protein_co	calcium-sensing receptor [Source:MGI Symbol;Acc:MGI:1351351]
ENSMUSG00000104010	Gm37366	36.04075	13.32853	1.431781	0.017635	TEC	predicted gene, 37366 [Source:MGI Symbol;Acc:MGI:5610594]
ENSMUSG00000035694	Caps2	13.82795	2.780543	2.351505	0.017877	protein_co	calcyphosphine 2 [Source:MGI Symbol;Acc:MGI:2441980]
ENSMUSG00000081597	Gm14506	1.644041	11.63947	-2.81154	0.018547	processed_	predicted gene 14506 [Source:MGI Symbol;Acc:MGI:3705552]
ENSMUSG00000025905	Oprk1	95.76665	22.17969	2.117727	0.01865	protein_co	opioid receptor, kappa 1 [Source:MGI Symbol;Acc:MGI:97439]
ENSMUSG00000031111	Igsf1	251.0074	91.17491	1.461655	0.018676	protein_co	immunoglobulin superfamily, member 1 [Source:MGI Symbol;Acc:I
ENSMUSG00000092492	B230208B08I	26.29859	4.717504	2.485705	0.018979	processed_	RIKEN cDNA B230208B08 gene [Source:MGI Symbol;Acc:MGI:2442
ENSMUSG00000039323	Igfbp2	1232.543	700.8928	0.814484	0.018982	protein_co	insulin-like growth factor binding protein 2 [Source:MGI Symbol;Ac
ENSMUSG00000020169	Best3	23.04736	2.718566	3.094908	0.018999	protein_co	bestrophin 3 [Source:MGI Symbol;Acc:MGI:3580298]
ENSMUSG00000021223	Papln	51.45215	17.79157	1.537665	0.0192	protein_co	papilin, proteoglycan-like sulfated glycoprotein [Source:MGI Symbo
ENSMUSG00000079070	Gm3985	21.57244	3.454853	2.664008	0.019315	lincRNA	predicted gene 3985 [Source:MGI Symbol;Acc:MGI:3782158]
ENSMUSG00000035451	Foxa1	16.56721	0.985141	4.078771	0.019441	protein_co	forkhead box A1 [Source:MGI Symbol;Acc:MGI:1347472]
ENSMUSG00000028717	Tal1	145.7325	41.78082	1.805277	0.020037	protein_co	T cell acute lymphocytic leukemia 1 [Source:MGI Symbol;Acc:MGI:
ENSMUSG00000047773	Ankfn1	35.14839	12.34978	1.516461	0.02006	protein_co	ankyrin-repeat and fibronectin type III domain containing 1 [Source
ENSMUSG00000036198	Arhgap36	95.77994	20.41683	2.233904	0.020134	protein_co	Rho GTPase activating protein 36 [Source:MGI Symbol;Acc:MGI:19
ENSMUSG00000025229	Pitx3	14.34981	1.279311	3.481275	0.020401	protein_co	paired-like homeodomain transcription factor 3 [Source:MGI Symb
ENSMUSG00000116755	CT025584.1	15.43094	0.911167	4.037171	0.020442	lincRNA	novel transcript
ENSMUSG00000024907	Gal	40.44236	13.68028	1.558852	0.020469	protein_co	galanin [Source:MGI Symbol;Acc:MGI:95637]
ENSMUSG00000069588	Gm11733	5.648228	0.29417	4.006735	0.020632	protein_co	predicted gene 11733 [Source:MGI Symbol;Acc:MGI:3650737]
ENSMUSG00000028270	Gbp2	87.2457	44.48782	0.972465	0.020661	protein_co	guanylate binding protein 2 [Source:MGI Symbol;Acc:MGI:102772]
ENSMUSG00000001901	Kcnh6	69.2882	29.4969	1.232907	0.020725	protein_co	potassium voltage-gated channel, subfamily H (eag-related), memb
ENSMUSG00000021506	Pitx1	11.49361	0.645654	4.148122	0.020867	protein_co	paired-like homeodomain transcription factor 1 [Source:MGI Symb
ENSMUSG00000036699	Zcchc12	1980.099	679.8627	1.542305	0.021511	protein_co	zinc finger, CCHC domain containing 12 [Source:MGI Symbol;Acc:M
ENSMUSG00000040181	Fmo1	183.5524	81.87586	1.163644	0.021805	protein_co	flavin containing monooxygenase 1 [Source:MGI Symbol;Acc:MGI:
ENSMUSG00000045915	Ccdc42	20.66002	5.80355	1.828933	0.022554	protein_co	coiled-coil domain containing 42 [Source:MGI Symbol;Acc:MGI:304
ENSMUSG00000112343	Sfta3-ps	15.79514	1.676111	3.251206	0.022956	processed_	surfactant associated 3, pseudogene [Source:MGI Symbol;Acc:MGI
ENSMUSG00000051354	Samd3	20.10856	6.25033	1.693242	0.023111	protein_co	sterile alpha motif domain containing 3 [Source:MGI Symbol;Acc:M

ENSMUSG00000050370	Ch25h	17.92675	4.411337	2.046042	0.023383	protein_co	cholesterol 25-hydroxylase [Source:MGI Symbol;Acc:MGI:1333869
ENSMUSG00000026247	Ecel1	282.5478	82.78365	1.772196	0.023687	protein_co	endothelin converting enzyme-like 1 [Source:MGI Symbol;Acc:MGI
ENSMUSG00000080242	Atp6v0c-ps2	26.91147	93.14939	-1.79157	0.024071	processed_	ATPase, H+ transporting, lysosomal V0 subunit C, pseudogene 2 [Sc
ENSMUSG00000046922	Gpr6	46.27486	9.904057	2.227709	0.024303	protein_co	G protein-coupled receptor 6 [Source:MGI Symbol;Acc:MGI:215524
ENSMUSG00000107017	Gm43196	6.629728	0.29417	4.231017	0.02432	TEC	predicted gene 43196 [Source:MGI Symbol;Acc:MGI:5663333]
ENSMUSG00000107909	2610017A05I	3.644459	13.44048	-1.87804	0.024822	TEC	RIKEN cDNA 2610017A05 gene [Source:MGI Symbol;Acc:MGI:1919
ENSMUSG00000068614	Actc1	5.787627	0.322827	4.0375	0.025133	protein_co	actin, alpha, cardiac muscle 1 [Source:MGI Symbol;Acc:MGI:87905]
ENSMUSG00000111765	Gm10635	8.12767	0.662314	3.630117	0.025397	lincRNA	predicted gene 10635 [Source:MGI Symbol;Acc:MGI:3641740]
ENSMUSG00000070084	Mir486	1.047234	12.27831	-3.57257	0.025441	miRNA	microRNA 486 [Source:MGI Symbol;Acc:MGI:3619423]
ENSMUSG00000046500	Fam19a4	17.8193	3.290246	2.443472	0.02546	protein_co	family with sequence similarity 19, member A4 [Source:MGI Symbo
ENSMUSG00000099759	1700030C10I	19.18369	5.186553	1.881987	0.02548	processed_	RIKEN cDNA 1700030C10 gene [Source:MGI Symbol;Acc:MGI:1916
ENSMUSG00000106832	Gm42632	12.40395	2.338425	2.416652	0.025694	TEC	predicted gene 42632 [Source:MGI Symbol;Acc:MGI:5662769]
ENSMUSG00000025316	Banp	667.8149	1095.093	-0.71376	0.025991	protein_co	BTG3 associated nuclear protein [Source:MGI Symbol;Acc:MGI:188
ENSMUSG00000066279	Chrna10	9.34932	0.322827	4.728242	0.026168	protein_co	cholinergic receptor, nicotinic, alpha polypeptide 10 [Source:MGI S
ENSMUSG00000023274	Cd4	25.6718	4.767484	2.44616	0.0262	protein_co	CD4 antigen [Source:MGI Symbol;Acc:MGI:88335]
ENSMUSG00000112466	Gm29674	46.03916	9.457276	2.281695	0.026212	lincRNA	predicted gene, 29674 [Source:MGI Symbol;Acc:MGI:5588833]
ENSMUSG00000024647	Cbln2	348.1619	159.7077	1.124054	0.026422	protein_co	cerebellin 2 precursor protein [Source:MGI Symbol;Acc:MGI:88282
ENSMUSG00000048602	Morc2b	21.47508	46.14339	-1.09849	0.02666	protein_co	microrchidia 2B [Source:MGI Symbol;Acc:MGI:3045293]
ENSMUSG00000031727	Pmfbp1	27.24189	6.030134	2.187782	0.026826	protein_co	polyamine modulated factor 1 binding protein 1 [Source:MGI Symb
ENSMUSG00000079497	Gm13420	7.418548	0.662314	3.507175	0.0271	protein_co	predicted gene 13420 [Source:MGI Symbol;Acc:MGI:3649917]
ENSMUSG00000090486	BC035947	91.87231	28.38393	1.698763	0.027143	protein_co	cDNA sequence BC035947 [Source:MGI Symbol;Acc:MGI:2652858]
ENSMUSG00000031789	Cngb1	26.24223	8.742312	1.580843	0.027217	protein_co	cyclic nucleotide gated channel beta 1 [Source:MGI Symbol;Acc:MG
ENSMUSG00000086905	Gm13716	53.13858	14.07681	1.914326	0.027255	antisense	predicted gene 13716 [Source:MGI Symbol;Acc:MGI:3650432]
ENSMUSG00000093460	Six3os1	70.87673	26.4543	1.428288	0.027664	processed_	SIX homeobox 3, opposite strand 1 [Source:MGI Symbol;Acc:MGI:1
ENSMUSG00000031492	Chrnb3	46.31351	10.35084	2.167572	0.028368	protein_co	cholinergic receptor, nicotinic, beta polypeptide 3 [Source:MGI Syn
ENSMUSG00000075224	Lrrc55	301.8861	90.55237	1.736306	0.028473	protein_co	leucine rich repeat containing 55 [Source:MGI Symbol;Acc:MGI:268
ENSMUSG00000021032	Ngb	202.3213	60.22063	1.748285	0.028521	protein_co	neuroglobin [Source:MGI Symbol;Acc:MGI:2151886]
ENSMUSG00000028778	Hcrtr1	118.2553	32.0506	1.885267	0.028676	protein_co	hypocretin (orexin) receptor 1 [Source:MGI Symbol;Acc:MGI:23856
ENSMUSG00000023945	Slc5a7	63.41056	13.34597	2.256436	0.028733	protein_co	solute carrier family 5 (choline transporter), member 7 [Source:MG
ENSMUSG00000020178	Adora2a	152.3418	51.27039	1.570719	0.028828	protein_co	adenosine A2a receptor [Source:MGI Symbol;Acc:MGI:99402]
ENSMUSG00000114452	A530001N23	6.312	0.322827	4.159464	0.028937	lincRNA	RIKEN cDNA A530001N23 gene [Source:MGI Symbol;Acc:MGI:3045
ENSMUSG00000032254	Kif23	20.73005	6.52784	1.663957	0.028962	protein_co	kinesin family member 23 [Source:MGI Symbol;Acc:MGI:1919069]
ENSMUSG00000020681	Ace	807.7867	427.7503	0.917422	0.029904	protein_co	angiotensin I converting enzyme (peptidyl-dipeptidase A) 1 [Source
ENSMUSG00000035783	Acta2	186.1513	111.5459	0.73865	0.029968	protein_co	actin, alpha 2, smooth muscle, aorta [Source:MGI Symbol;Acc:MGI

ENSMUSG00000031210	Gpr165	91.32978	32.10041	1.515181	0.030741	protein_co	G protein-coupled receptor 165 [Source:MGI Symbol;Acc:MGI:192
ENSMUSG00000029112	Nkx1-1	9.758591	0.616997	3.945313	0.031118	protein_co	NK1 homeobox 1 [Source:MGI Symbol;Acc:MGI:109346]
ENSMUSG00000043659	Npsr1	20.57725	3.890583	2.398451	0.031136	protein_co	neuropeptide S receptor 1 [Source:MGI Symbol;Acc:MGI:2441738]
ENSMUSG00000078952	Lncenc1	17.17044	3.352223	2.372122	0.031145	lincRNA	long non-coding RNA, embryonic stem cells expressed 1 [Source:M
ENSMUSG00000021685	Otp	77.04456	13.21468	2.548812	0.031287	protein_co	orthopedia homeobox [Source:MGI Symbol;Acc:MGI:99835]
ENSMUSG00000065746	Gm25005	3.686458	13.38145	-1.86941	0.03204	snRNA	predicted gene, 25005 [Source:MGI Symbol;Acc:MGI:5454782]
ENSMUSG00000106734	Gm20559	52.30335	23.80798	1.139633	0.032558	processed_	predicted gene, 20559 [Source:MGI Symbol;Acc:MGI:5295666]
ENSMUSG00000021803	Cdhr1	579.0477	54.98451	3.396297	0.032822	protein_co	cadherin-related family member 1 [Source:MGI Symbol;Acc:MGI:2
ENSMUSG00000109917	Gm45671	9.003556	1.104431	3.11188	0.033007	lincRNA	predicted gene 45671 [Source:MGI Symbol;Acc:MGI:5791507]
ENSMUSG00000020007	Il20ra	16.96267	3.928565	2.108034	0.033064	protein_co	interleukin 20 receptor, alpha [Source:MGI Symbol;Acc:MGI:36050
ENSMUSG00000047507	Baiap3	1468.08	486.4674	1.593746	0.033147	protein_co	BAI1-associated protein 3 [Source:MGI Symbol;Acc:MGI:2685783]
ENSMUSG00000086308	G630016G05	64.50563	18.01444	1.841461	0.033292	lincRNA	RIKEN cDNA G630016G05 gene [Source:MGI Symbol;Acc:MGI:360
ENSMUSG00000068452	Duox2	9.031592	0.322827	4.678378	0.033353	protein_co	dual oxidase 2 [Source:MGI Symbol;Acc:MGI:3036280]
ENSMUSG00000022425	Enpp2	11082.04	5171.314	1.099656	0.033368	protein_co	ectonucleotide pyrophosphatase/phosphodiesterase 2 [Source:MG
ENSMUSG00000073805	Fam196a	151.9594	62.51348	1.280168	0.033645	protein_co	family with sequence similarity 196, member A [Source:MGI Symb
ENSMUSG00000026778	Prkcq	258.8815	143.7761	0.848622	0.033847	protein_co	protein kinase C, theta [Source:MGI Symbol;Acc:MGI:97601]
ENSMUSG00000103200	Gm37328	5.197801	17.40625	-1.73687	0.034012	sense_intr	predicted gene, 37328 [Source:MGI Symbol;Acc:MGI:5610556]
ENSMUSG00000021647	Cartpt	209.3507	47.20655	2.148276	0.034015	protein_co	CART prepropeptide [Source:MGI Symbol;Acc:MGI:1351330]
ENSMUSG00000049134	Nrap	26.09977	9.030873	1.542715	0.034037	protein_co	nebulin-related anchoring protein [Source:MGI Symbol;Acc:MGI:1
ENSMUSG00000031130	Brs3	4.189216	0	4.541845	0.034053	protein_co	bombesin-like receptor 3 [Source:MGI Symbol;Acc:MGI:1100501]
ENSMUSG00000100548	Gm29585	0.321078	5.503771	-3.93192	0.034108	antisense	predicted gene 29585 [Source:MGI Symbol;Acc:MGI:5580291]
ENSMUSG00000028640	Tfap2c	8.260087	0.322827	4.549117	0.034147	protein_co	transcription factor AP-2, gamma [Source:MGI Symbol;Acc:MGI:10
ENSMUSG00000021611	Tert	36.11776	13.54484	1.423618	0.034168	protein_co	telomerase reverse transcriptase [Source:MGI Symbol;Acc:MGI:12
ENSMUSG00000000392	Fap	47.73322	17.86554	1.42333	0.03427	protein_co	fibroblast activation protein [Source:MGI Symbol;Acc:MGI:109608
ENSMUSG00000101578	Vmn1r206	14.38762	1.104431	3.761989	0.034356	protein_co	vomeronasal 1 receptor 206 [Source:MGI Symbol;Acc:MGI:215966
ENSMUSG00000062200	Vmn2r7	0	5.59268	-4.92497	0.034775	protein_co	vomeronasal 2, receptor 7 [Source:MGI Symbol;Acc:MGI:2441693]
ENSMUSG00000017817	Jph2	35.16962	9.570445	1.866883	0.035027	protein_co	junctophilin 2 [Source:MGI Symbol;Acc:MGI:1891496]
ENSMUSG00000024565	Sall3	172.9548	54.07706	1.676289	0.035041	protein_co	spalt like transcription factor 3 [Source:MGI Symbol;Acc:MGI:1092
ENSMUSG00000036867	Smad6	85.08011	45.75859	0.89859	0.035048	protein_co	SMAD family member 6 [Source:MGI Symbol;Acc:MGI:1336883]
ENSMUSG00000108010	Gm38708	12.41094	2.441056	2.381458	0.035387	lincRNA	predicted gene, 38708 [Source:MGI Symbol;Acc:MGI:5621593]
ENSMUSG00000099907	Gm10421	17.21886	3.613073	2.255523	0.035408	processed_	predicted gene 10421 [Source:MGI Symbol;Acc:MGI:3642601]
ENSMUSG00000097385	Gm26814	13.03148	35.44123	-1.43954	0.03548	TEC	predicted gene, 26814 [Source:MGI Symbol;Acc:MGI:5477308]
ENSMUSG00000060985	Tdrd5	65.71254	31.75497	1.046932	0.036028	protein_co	tudor domain containing 5 [Source:MGI Symbol;Acc:MGI:2684949]
ENSMUSG00000058152	Chsy3	65.80207	16.68342	1.978472	0.036408	protein_co	chondroitin sulfate synthase 3 [Source:MGI Symbol;Acc:MGI:19261

ENSMUSG00000038257	Glra3	187.7841	58.05596	1.695812	0.036997	protein_co	glycine receptor, alpha 3 subunit [Source:MGI Symbol;Acc:MGI:957
ENSMUSG00000116250	Gm34939	12.45265	2.603938	2.251676	0.03719	antisense	predicted gene, 34939 [Source:MGI Symbol;Acc:MGI:5594098]
ENSMUSG00000012819	Cdh23	138.4386	54.6163	1.344024	0.037855	protein_co	cadherin 23 (otocadherin) [Source:MGI Symbol;Acc:MGI:1890219]
ENSMUSG00000107207	Gm42897	1.410311	9.065139	-2.72519	0.037967	TEC	predicted gene 42897 [Source:MGI Symbol;Acc:MGI:5663034]
ENSMUSG00000034107	Ano7	16.77541	3.821272	2.128347	0.038466	protein_co	anoctamin 7 [Source:MGI Symbol;Acc:MGI:3052714]
ENSMUSG00000031075	Ano1	79.18024	41.74621	0.918394	0.038652	protein_co	anoctamin 1, calcium activated chloride channel [Source:MGI Symb
ENSMUSG00000048004	Tmem196	217.2561	107.2337	1.020399	0.038804	protein_co	transmembrane protein 196 [Source:MGI Symbol;Acc:MGI:268537
ENSMUSG00000047180	Neurl3	36.70843	15.3424	1.251547	0.038971	protein_co	neuralized E3 ubiquitin protein ligase 3 [Source:MGI Symbol;Acc:M
ENSMUSG00000029798	Herc6	101.4247	50.68523	0.999599	0.039003	protein_co	hect domain and RLD 6 [Source:MGI Symbol;Acc:MGI:1914388]
ENSMUSG00000046618	Olfml2a	112.3846	58.29696	0.948049	0.039519	protein_co	olfactomedin-like 2A [Source:MGI Symbol;Acc:MGI:2444741]
ENSMUSG00000022603	Mroh4	4.642993	0.322827	3.724383	0.040033	protein_co	maestro heat-like repeat family member 4 [Source:MGI Symbol;Ac
ENSMUSG00000078350	Smim1	311.6587	155.4883	1.003618	0.040076	protein_co	small integral membrane protein 1 [Source:MGI Symbol;Acc:MGI:1
ENSMUSG00000038677	Scube3	155.9196	72.45387	1.107085	0.040201	protein_co	signal peptide, CUB domain, EGF-like 3 [Source:MGI Symbol;Acc:M
ENSMUSG00000027820	Mme	129.8319	47.92454	1.439729	0.040625	protein_co	membrane metallo endopeptidase [Source:MGI Symbol;Acc:MGI:9
ENSMUSG00000020374	Rasgef1c	212.2506	114.4648	0.890285	0.040673	protein_co	RasGEF domain family, member 1C [Source:MGI Symbol;Acc:MGI:1
ENSMUSG00000030223	Ptpro	766.7649	242.9015	1.658131	0.040683	protein_co	protein tyrosine phosphatase, receptor type, O [Source:MGI Symbo
ENSMUSG00000043648	Pld6	46.62246	14.78566	1.651284	0.0407	protein_co	phospholipase D family, member 6 [Source:MGI Symbol;Acc:MGI:2
ENSMUSG00000105265	Sox2ot	861.0365	464.8129	0.889511	0.04116	processed_	SOX2 overlapping transcript (non-protein coding) [Source:MGI Sym
ENSMUSG00000097174	Gm4890	25.99024	9.898448	1.410627	0.041409	bidirection	predicted gene 4890 [Source:MGI Symbol;Acc:MGI:3779445]
ENSMUSG00000021763	BC067074	53.46696	22.84684	1.227069	0.041592	protein_co	cDNA sequence BC067074 [Source:MGI Symbol;Acc:MGI:3040697]
ENSMUSG00000066113	Adamtsl1	146.0438	77.06461	0.923064	0.041692	protein_co	ADAMTS-like 1 [Source:MGI Symbol;Acc:MGI:1924989]
ENSMUSG00000031665	Sall1	477.3805	196.8227	1.278106	0.04191	protein_co	spalt like transcription factor 1 [Source:MGI Symbol;Acc:MGI:1889
ENSMUSG00000058741	Prr19	7.246919	1.307967	2.475383	0.042648	protein_co	proline rich 19 [Source:MGI Symbol;Acc:MGI:3648539]
ENSMUSG00000067261	Foxd3	25.23404	0.29417	6.165051	0.042681	protein_co	forkhead box D3 [Source:MGI Symbol;Acc:MGI:1347473]
ENSMUSG00000105891	A230001M10	61.16165	25.92276	1.239135	0.04282	lincRNA	RIKEN cDNA A230001M10 gene [Source:MGI Symbol;Acc:MGI:244
ENSMUSG00000047586	Nccrp1	9.111007	1.544824	2.524057	0.043094	protein_co	non-specific cytotoxic cell receptor protein 1 homolog (zebrafish) [
ENSMUSG00000021219	Rgs6	859.1065	429.8309	0.999415	0.043115	protein_co	regulator of G-protein signaling 6 [Source:MGI Symbol;Acc:MGI:13
ENSMUSG00000113157	Gm48693	6.133952	0.616997	3.260235	0.043149	processed_	predicted gene, 48693 [Source:MGI Symbol;Acc:MGI:6098323]
ENSMUSG00000005611	Mrvi1	68.98698	35.84132	0.950708	0.043167	protein_co	MRV integration site 1 [Source:MGI Symbol;Acc:MGI:1338023]
ENSMUSG00000057315	Arhgap24	207.1097	91.74669	1.176013	0.043735	protein_co	Rho GTPase activating protein 24 [Source:MGI Symbol;Acc:MGI:19
ENSMUSG00000025584	Pde8a	440.3292	253.1648	0.799333	0.044	protein_co	phosphodiesterase 8A [Source:MGI Symbol;Acc:MGI:1277116]
ENSMUSG00000064354	mt-Co2	725.874	1875.124	-1.36935	0.044031	protein_co	mitochondrially encoded cytochrome c oxidase II [Source:MGI Sym
ENSMUSG00000020713	Gh	701.599	38.51463	4.186904	0.044032	protein_co	growth hormone [Source:MGI Symbol;Acc:MGI:95707]
ENSMUSG00000106375	Gm43361	0.638807	5.898847	-3.16774	0.044077	TEC	predicted gene 43361 [Source:MGI Symbol;Acc:MGI:5663498]

ENSMUSG00000097817	Gm26810	3.595759	0	4.319697	0.044508	antisense	predicted gene, 26810 [Source:MGI Symbol;Acc:MGI:5477304]
ENSMUSG00000086272	BC039966	85.15061	18.43404	2.206653	0.044858	antisense	cDNA sequence BC039966 [Source:MGI Symbol;Acc:MGI:3039570]
ENSMUSG00000019888	Mgat4c	133.1006	59.99163	1.150282	0.045335	protein_co	MGAT4 family, member C [Source:MGI Symbol;Acc:MGI:1914819]
ENSMUSG00000110397	Gm45540	4.86304	0.368144	3.78275	0.046052	TEC	predicted gene 45540 [Source:MGI Symbol;Acc:MGI:5791376]
ENSMUSG00000042761	Mrap2	407.1296	152.0066	1.422037	0.046519	protein_co	melanocortin 2 receptor accessory protein 2 [Source:MGI Symbol;/
ENSMUSG00000026196	Bard1	19.98228	7.440733	1.442303	0.04653	protein_co	BRCA1 associated RING domain 1 [Source:MGI Symbol;Acc:MGI:13
ENSMUSG00000028864	Hgf	28.06555	5.402086	2.367777	0.046784	protein_co	hepatocyte growth factor [Source:MGI Symbol;Acc:MGI:96079]
ENSMUSG00000107859	Gm30731	39.92257	18.77671	1.097713	0.046976	lincRNA	predicted gene, 30731 [Source:MGI Symbol;Acc:MGI:5589890]
ENSMUSG00000074939	Chrm5	21.39439	3.228269	2.723617	0.046999	protein_co	cholinergic receptor, muscarinic 5 [Source:MGI Symbol;Acc:MGI:1(
ENSMUSG00000073421	H2-Ab1	75.60164	36.78236	1.042911	0.047004	protein_co	histocompatibility 2, class II antigen A, beta 1 [Source:MGI Symbol;
ENSMUSG00000028172	Tacr3	89.53043	32.21374	1.475884	0.047159	protein_co	tachykinin receptor 3 [Source:MGI Symbol;Acc:MGI:892968]
ENSMUSG00000045932	Ifit2	179.8859	112.5796	0.675059	0.047243	protein_co	interferon-induced protein with tetratricopeptide repeats 2 [Sourc
ENSMUSG00000085433	Gm16001	61.25613	32.95642	0.896805	0.047371	lincRNA	predicted gene 16001 [Source:MGI Symbol;Acc:MGI:3802119]
ENSMUSG00000078252	Krtap17-1	4.179165	0	4.537883	0.047671	protein_co	keratin associated protein 17-1 [Source:MGI Symbol;Acc:MGI:1925
ENSMUSG00000086126	Evx1os	11.54231	1.544824	2.879508	0.047681	antisense	even skipped homeotic gene 1, opposite strand [Source:MGI Symb
ENSMUSG00000025938	Slco5a1	116.2787	45.25355	1.361037	0.047723	protein_co	solute carrier organic anion transporter family, member 5A1 [Sour
ENSMUSG00000024084	Qpct	199.6241	85.59639	1.221093	0.048047	protein_co	glutaminyl-peptide cyclotransferase (glutaminyl cyclase) [Source:M
ENSMUSG00000085008	Dbhos	6.127252	0.368144	4.121323	0.048064	antisense	dopamine beta hydroxylase, opposite strand [Source:MGI Symbol;/
ENSMUSG00000024871	Doc2g	379.3256	178.0272	1.090443	0.048948	protein_co	double C2, gamma [Source:MGI Symbol;Acc:MGI:1926250]
ENSMUSG00000073154	9330158H04I	39.37319	16.53013	1.251847	0.049706	lincRNA	RIKEN cDNA 9330158H04 gene [Source:MGI Symbol;Acc:MGI:2442
ENSMUSG00000109473	B930025P03I	14.19494	3.646392	1.974408	0.049713	lincRNA	RIKEN cDNA B930025P03 gene [Source:MGI Symbol;Acc:MGI:2443
ENSMUSG00000039684	Gm5422	9.988126	1.353284	2.897469	0.04983	transcribe	predicted pseudogene 5422 [Source:MGI Symbol;Acc:MGI:364341
ENSMUSG00000034755	Pcdh11x	185.7843	74.35527	1.321398	0.049895	protein_co	protocadherin 11 X-linked [Source:MGI Symbol;Acc:MGI:2442849]

HF1GvsHFD

gene_id	gene_name	HF1G_fpkr	HFD_fpkm	log2FoldCh	pvalue	gene_bioty	gene_description
ENSMUSG00000056648	Hoxb8	28.63292	0.345477	6.39235	0.000549	protein_co	homeobox B8 [Source:MGI Symbol;Acc:MGI:96189]
ENSMUSG00000000690	Hoxb6	40.319	1.21437	5.030096	0.001041	protein_co	homeobox B6 [Source:MGI Symbol;Acc:MGI:96187]
ENSMUSG00000044285	Gm1821	270.3343	92.10169	1.552506	0.001176	transcribe	predicted gene 1821 [Source:MGI Symbol;Acc:MGI:3037679]
ENSMUSG00000019874	Fabp7	248.0651	787.7392	-1.6663	0.001236	protein_co	fatty acid binding protein 7, brain [Source:MGI Symbol;Acc:MGI:10
ENSMUSG00000020713	Gh	4.910129	668.7009	-7.08542	0.001434	protein_co	growth hormone [Source:MGI Symbol;Acc:MGI:95707]
ENSMUSG00000099342	Gm18180	4.954174	24.7798	-2.33967	0.00241	processed_	predicted gene, 18180 [Source:MGI Symbol;Acc:MGI:5010365]
ENSMUSG00000021250	Fos	101.913	316.0442	-1.63453	0.002476	protein_co	FBJ osteosarcoma oncogene [Source:MGI Symbol;Acc:MGI:95574]
ENSMUSG00000053441	Adamts19	5.108691	66.57302	-3.70072	0.002738	protein_co	a disintegrin-like and metallopeptidase (reprolysin type) with thror

ENSMUSG00000063935	Zar1	1.681013	19.16337	-3.49515	0.00332	protein_co	zygote arrest 1 [Source:MGI Symbol;Acc:MGI:2180337]
ENSMUSG00000051985	Igfn1	60.35827	259.5613	-2.10487	0.004452	protein_co	immunoglobulin-like and fibronectin type III domain containing 1 [S
ENSMUSG00000031491	Chrna6	6.931198	100.674	-3.85476	0.004488	protein_co	cholinergic receptor, nicotinic, alpha polypeptide 6 [Source:MGI Sy
ENSMUSG00000036480	Prss56	3.748467	34.61264	-3.20072	0.004527	protein_co	protease, serine 56 [Source:MGI Symbol;Acc:MGI:1916703]
ENSMUSG00000108993	Gm44846	0	6.321678	-5.05771	0.005315	TEC	predicted gene 44846 [Source:MGI Symbol;Acc:MGI:5753422]
ENSMUSG00000027485	Bpifb1	6.787365	0	5.271087	0.006706	protein_co	BPI fold containing family B, member 1 [Source:MGI Symbol;Acc:M
ENSMUSG00000028197	Col24a1	37.75693	167.8478	-2.15049	0.007286	protein_co	collagen, type XXIV, alpha 1 [Source:MGI Symbol;Acc:MGI:1918605
ENSMUSG00000012819	Cdh23	42.27023	131.8788	-1.64179	0.01034	protein_co	cadherin 23 (otocadherin) [Source:MGI Symbol;Acc:MGI:1890219]
ENSMUSG00000116755	CT025584.1	0.320253	14.68501	-5.3082	0.010547	lincRNA	novel transcript
ENSMUSG00000110086	Gm45623	21.68115	66.11	-1.60908	0.010568	protein_co	predicted gene 45623 [Source:MGI Symbol;Acc:MGI:5791459]
ENSMUSG00000060284	Sp7	31.50872	114.6882	-1.86082	0.011476	protein_co	Sp7 transcription factor 7 [Source:MGI Symbol;Acc:MGI:2153568]
ENSMUSG00000031167	Rbm3	2347.569	1285.283	0.869178	0.011699	protein_co	RNA binding motif (RNP1, RRM) protein 3 [Source:MGI Symbol;Acc
ENSMUSG00000018166	Erbb3	213.0063	371.0472	-0.80039	0.011802	protein_co	erb-b2 receptor tyrosine kinase 3 [Source:MGI Symbol;Acc:MGI:95
ENSMUSG00000105504	Gbp5	98.2162	36.17942	1.444242	0.012152	protein_co	guanylate binding protein 5 [Source:MGI Symbol;Acc:MGI:2429943
ENSMUSG00000113722	Snhg10	47.31093	97.41196	-1.0442	0.012188	lincRNA	small nucleolar RNA host gene 10 [Source:MGI Symbol;Acc:MGI:19
ENSMUSG00000109297	Gm31522	0	5.623278	-4.8826	0.012497	lincRNA	predicted gene, 31522 [Source:MGI Symbol;Acc:MGI:5590681]
ENSMUSG00000073154	9330158H04I	10.69269	37.50526	-1.80179	0.012588	lincRNA	RIKEN cDNA 9330158H04 gene [Source:MGI Symbol;Acc:MGI:2442
ENSMUSG00000050473	Slc35d3	23.94614	73.15179	-1.60656	0.012676	protein_co	solute carrier family 35, member D3 [Source:MGI Symbol;Acc:MGI:
ENSMUSG00000115624	Gm49204	20.92573	58.02716	-1.4732	0.012936	TEC	predicted gene, 49204 [Source:MGI Symbol;Acc:MGI:6118653]
ENSMUSG00000076498	Trbc2	8.309421	32.97256	-1.99119	0.01333	TR_C_gene	T cell receptor beta, constant 2 [Source:MGI Symbol;Acc:MGI:4835
ENSMUSG00000020007	Il20ra	3.147566	16.14808	-2.36318	0.013864	protein_co	interleukin 20 receptor, alpha [Source:MGI Symbol;Acc:MGI:36050
ENSMUSG00000025229	Pitx3	0.667089	13.67694	-4.33845	0.013938	protein_co	paired-like homeodomain transcription factor 3 [Source:MGI Symb
ENSMUSG00000026308	Klhl30	3.302083	29.99642	-3.16701	0.014215	protein_co	kelch-like 30 [Source:MGI Symbol;Acc:MGI:1918038]
ENSMUSG00000087075	Lbhd2	19.26422	102.7035	-2.41129	0.01481	protein_co	LBH domain containing 2 [Source:MGI Symbol;Acc:MGI:2685744]
ENSMUSG00000084390	Gm15425	7.691057	0.651287	3.593534	0.015368	processed_	predicted gene 15425 [Source:MGI Symbol;Acc:MGI:3705642]
ENSMUSG00000095041	AC149090.1	7725.331	12451.89	-0.68867	0.016422	protein_coding	
ENSMUSG00000079363	Gbp4	166.5749	41.15743	2.017806	0.016763	protein_co	guanylate binding protein 4 [Source:MGI Symbol;Acc:MGI:97072]
ENSMUSG00000080242	Atp6v0c-ps2	103.8517	25.63096	2.018152	0.016918	processed_	ATPase, H+ transporting, lysosomal V0 subunit C, pseudogene 2 [So
ENSMUSG00000111193	Gm35288	0.320253	6.104072	-4.04224	0.017381	lincRNA	predicted gene, 35288 [Source:MGI Symbol;Acc:MGI:5594447]
ENSMUSG00000097488	4732487G21I	59.39097	32.05267	0.890565	0.017441	lincRNA	RIKEN cDNA 4732487G21 gene [Source:MGI Symbol;Acc:MGI:2441
ENSMUSG00000115354	Gm49083	0.346836	11.57572	-4.96434	0.017603	lincRNA	predicted gene, 49083 [Source:MGI Symbol;Acc:MGI:6118469]
ENSMUSG00000041679	Lrrc29	101.4423	51.93644	0.970018	0.017786	protein_co	leucine rich repeat containing 29 [Source:MGI Symbol;Acc:MGI:244
ENSMUSG00000090698	Apold1	47.82117	121.427	-1.34474	0.019705	protein_co	apolipoprotein L domain containing 1 [Source:MGI Symbol;Acc:MG
ENSMUSG00000045613	Chrm2	130.7983	299.0951	-1.19291	0.020267	protein_co	cholinergic receptor, muscarinic 2, cardiac [Source:MGI Symbol;Acc

ENSMUSG00000112975	Gm21297	0.320253	6.177794	-4.06698	0.020347	processed_	predicted gene, 21297 [Source:MGI Symbol;Acc:MGI:5434652]
ENSMUSG00000110016	Gm20751	65.91358	35.87372	0.875257	0.020937	lincRNA	predicted gene, 20751 [Source:MGI Symbol;Acc:MGI:5434107]
ENSMUSG00000071665	Foxr2	30.54328	11.47457	1.412818	0.021174	protein_co	forkhead box R2 [Source:MGI Symbol;Acc:MGI:3511682]
ENSMUSG00000047606	Ankrd34c	53.92388	140.031	-1.3754	0.021584	protein_co	ankyrin repeat domain 34C [Source:MGI Symbol;Acc:MGI:2685617
ENSMUSG00000090084	Srpx	2.387707	10.95706	-2.18853	0.022385	protein_co	sushi-repeat-containing protein [Source:MGI Symbol;Acc:MGI:185
ENSMUSG00000096225	Lhx8	0.686891	16.09386	-4.55244	0.022651	protein_co	LIM homeobox protein 8 [Source:MGI Symbol;Acc:MGI:1096343]
ENSMUSG00000057836	Xlr3a	15.47986	36.95899	-1.25821	0.022751	protein_co	X-linked lymphocyte-regulated 3A [Source:MGI Symbol;Acc:MGI:1
ENSMUSG00000028860	Sytl1	17.37246	39.65937	-1.19351	0.022846	protein_co	synaptotagmin-like 1 [Source:MGI Symbol;Acc:MGI:1933365]
ENSMUSG00000067455	Hist1h4j	3.120984	12.90827	-2.05628	0.022872	protein_co	histone cluster 1, H4j [Source:MGI Symbol;Acc:MGI:2448436]
ENSMUSG00000111017	Gm48314	0	4.204661	-4.46542	0.023312	TEC	predicted gene, 48314 [Source:MGI Symbol;Acc:MGI:6097766]
ENSMUSG00000099719	Gm28802	4.095303	0	4.54828	0.023587	unprocess	predicted gene 28802 [Source:MGI Symbol;Acc:MGI:5579508]
ENSMUSG00000035179	Ppp1r32	51.66391	23.61693	1.133247	0.024393	protein_co	protein phosphatase 1, regulatory subunit 32 [Source:MGI Symbol;
ENSMUSG00000017309	Cd300lg	10.48501	2.256714	2.232134	0.024676	protein_co	CD300 molecule like family member G [Source:MGI Symbol;Acc:M
ENSMUSG00000045471	Hcrt	2.394487	16.19684	-2.75908	0.025079	protein_co	hypocretin [Source:MGI Symbol;Acc:MGI:1202306]
ENSMUSG00000038491	Cdk19os	0.346836	5.345611	-3.85533	0.025239	antisense	cyclin-dependent kinase 19, opposite strand [Source:MGI Symbol;
ENSMUSG00000027849	Syt6	232.9937	551.9873	-1.24354	0.025635	protein_co	synaptotagmin VI [Source:MGI Symbol;Acc:MGI:1859544]
ENSMUSG00000021303	Gng4	373.2055	1213.663	-1.70137	0.025848	protein_co	guanine nucleotide binding protein (G protein), gamma 4 [Source:
ENSMUSG00000025094	Slc18a2	177.9385	590.801	-1.73112	0.026264	protein_co	solute carrier family 18 (vesicular monoamine), member 2 [Source:
ENSMUSG00000024211	Grm8	100.0067	203.632	-1.02662	0.026322	protein_co	glutamate receptor, metabotropic 8 [Source:MGI Symbol;Acc:MGI:
ENSMUSG00000064179	Tnnt1	21.71739	82.88769	-1.93171	0.02673	protein_co	troponin T1, skeletal, slow [Source:MGI Symbol;Acc:MGI:1333868]
ENSMUSG00000042918	Mamstr	85.138	45.78863	0.896407	0.026864	protein_co	MEF2 activating motif and SAP domain containing transcriptional r
ENSMUSG00000071547	Nt5dc2	224.3558	134.549	0.738381	0.026935	protein_co	5'-nucleotidase domain containing 2 [Source:MGI Symbol;Acc:MGI
ENSMUSG00000097303	3110083C13I	6.456428	19.04467	-1.55817	0.026998	bidirection	RIKEN cDNA 3110083C13 gene [Source:MGI Symbol;Acc:MGI:1920
ENSMUSG00000053368	Rxfp2	1.720618	9.961833	-2.53859	0.027021	protein_co	relaxin/insulin-like family peptide receptor 2 [Source:MGI Symbol;
ENSMUSG00000017832	Hspb9	4.801997	0.30581	3.815262	0.027408	protein_co	heat shock protein, alpha-crystallin-related, B9 [Source:MGI Symbo
ENSMUSG00000107749	Gm44321	1.033727	7.061168	-2.77124	0.027711	TEC	predicted gene, 44321 [Source:MGI Symbol;Acc:MGI:5690713]
ENSMUSG00000030549	Rhcg	3.880842	24.32767	-2.65382	0.02812	protein_co	Rhesus blood group-associated C glycoprotein [Source:MGI Symbo
ENSMUSG00000115783	Bc1	9.983655	34.26372	-1.78227	0.028311	lincRNA	brain cytoplasmic RNA 1 [Source:MGI Symbol;Acc:MGI:104905]
ENSMUSG00000100287	Gm28068	3.741688	0	4.418553	0.028683	lincRNA	predicted gene 28068 [Source:MGI Symbol;Acc:MGI:5578774]
ENSMUSG00000092574	2810047C21I	5.029481	0.345477	3.886858	0.028746	transcribe	RIKEN cDNA 2810047C21 gene 1 [Source:MGI Symbol;Acc:MGI:191
ENSMUSG00000056025	Clca3a1	5.247845	30.47721	-2.53966	0.029005	protein_co	chloride channel accessory 3A1 [Source:MGI Symbol;Acc:MGI:1316
ENSMUSG00000073409	H2-Q6	125.785	37.30405	1.755954	0.029063	protein_co	histocompatibility 2, Q region locus 6 [Source:MGI Symbol;Acc:MG
ENSMUSG00000023274	Cd4	4.369171	24.46504	-2.48043	0.029223	protein_co	CD4 antigen [Source:MGI Symbol;Acc:MGI:88335]
ENSMUSG00000030098	Grip2	418.8549	827.8603	-0.98252	0.029309	protein_co	glutamate receptor interacting protein 2 [Source:MGI Symbol;Acc:

ENSMUSG00000107499	Ccdc142	0.686891	6.299751	-3.19897	0.030604	protein_co	coiled-coil domain containing 142 [Source:MGI Symbol;Acc:MGI:30
ENSMUSG00000064364	mt-Th	158.2371	71.07074	1.156907	0.030874	Mt_tRNA	mitochondrially encoded tRNA histidine [Source:MGI Symbol;Acc:M
ENSMUSG00000032125	Robo4	211.5625	135.2668	0.644832	0.031612	protein_co	roundabout guidance receptor 4 [Source:MGI Symbol;Acc:MGI:192
ENSMUSG00000035451	Foxa1	0.640507	15.791	-4.58636	0.033367	protein_co	forkhead box A1 [Source:MGI Symbol;Acc:MGI:1347472]
ENSMUSG00000102222	Pcdhga10	5.462842	0.345477	4.002127	0.033369	protein_co	protocadherin gamma subfamily A, 10 [Source:MGI Symbol;Acc:MG
ENSMUSG00000099964	Gm29514	25.18965	47.88057	-0.92973	0.033397	antisense	predicted gene 29514 [Source:MGI Symbol;Acc:MGI:5580220]
ENSMUSG00000038550	Ciart	408.2729	242.0425	0.754354	0.033421	protein_co	circadian associated repressor of transcription [Source:MGI Symbo
ENSMUSG00000029019	Nppb	4.762392	0.30581	3.805203	0.033878	protein_co	natriuretic peptide type B [Source:MGI Symbol;Acc:MGI:97368]
ENSMUSG00000027233	Patl2	0.693671	8.313782	-3.59307	0.034766	protein_co	protein associated with topoisomerase II homolog 2 (yeast) [Sourc
ENSMUSG00000103477	5930409G06I	20.6542	56.16112	-1.44263	0.035091	TEC	RIKEN cDNA 5930409G06 gene [Source:MGI Symbol;Acc:MGI:1924
ENSMUSG00000026489	Coq8a	1097.809	748.5378	0.55248	0.035327	protein_co	coenzyme Q8A [Source:MGI Symbol;Acc:MGI:1914676]
ENSMUSG00000045534	Kcna5	35.68768	83.7591	-1.23056	0.035489	protein_co	potassium voltage-gated channel, shaker-related subfamily, memb
ENSMUSG00000061086	Myl4	27.88171	68.59869	-1.30063	0.03559	protein_co	myosin, light polypeptide 4 [Source:MGI Symbol;Acc:MGI:97267]
ENSMUSG00000005892	Trh	29.08274	156.3334	-2.42819	0.036888	protein_co	thyrotropin releasing hormone [Source:MGI Symbol;Acc:MGI:9882
ENSMUSG00000025652	Tmem89	0	3.994501	-4.39983	0.037004	protein_co	transmembrane protein 89 [Source:MGI Symbol;Acc:MGI:1916634
ENSMUSG00000091955	Gm9844	49.603	124.3898	-1.32842	0.037137	protein_co	predicted pseudogene 9844 [Source:MGI Symbol;Acc:MGI:370428
ENSMUSG00000083226	Gm7831	7.589704	0.91743	3.0199	0.037296	processed_	predicted gene 7831 [Source:MGI Symbol;Acc:MGI:3643841]
ENSMUSG00000037034	Pax1	6.595582	0.957097	2.787667	0.037583	protein_co	paired box 1 [Source:MGI Symbol;Acc:MGI:97485]
ENSMUSG00000045010	Gm4779	6.096033	22.07586	-1.85194	0.03774	protein_co	predicted gene 4779 [Source:MGI Symbol;Acc:MGI:3646776]
ENSMUSG00000098202	B830012L14I	1.727398	8.321119	-2.26947	0.037909	lincRNA	RIKEN cDNA B830012L14 gene [Source:MGI Symbol;Acc:MGI:2443
ENSMUSG00000024670	Cd6	2.087257	10.81288	-2.37523	0.037943	protein_co	CD6 antigen [Source:MGI Symbol;Acc:MGI:103566]
ENSMUSG00000041616	Nppa	1.033727	9.505813	-3.19503	0.0386	protein_co	natriuretic peptide type A [Source:MGI Symbol;Acc:MGI:97367]
ENSMUSG00000062200	Vmn2r7	5.336711	0	4.931613	0.038766	protein_co	vomeronasal 2, receptor 7 [Source:MGI Symbol;Acc:MGI:2441693]
ENSMUSG00000101578	Vmn1r206	0.96076	13.70018	-3.79621	0.039896	protein_co	vomeronasal 1 receptor 206 [Source:MGI Symbol;Acc:MGI:215966
ENSMUSG00000035582	Gdpd4	4.322787	32.99262	-2.92264	0.04044	protein_co	glycerophosphodiester phosphodiesterase domain containing 4 [Sc
ENSMUSG00000050211	Pla2g4e	270.1998	139.2248	0.957768	0.040568	protein_co	phospholipase A2, group IVE [Source:MGI Symbol;Acc:MGI:191914
ENSMUSG00000110236	Gm40493	3.074599	12.68007	-2.041	0.040713	antisense	predicted gene, 40493 [Source:MGI Symbol;Acc:MGI:5623378]
ENSMUSG00000115284	Gm34678	7.503178	1.562804	2.254359	0.040937	antisense	predicted gene, 34678 [Source:MGI Symbol;Acc:MGI:5593837]
ENSMUSG00000107089	Gm43443	2.087257	11.02753	-2.40535	0.041013	TEC	predicted gene 43443 [Source:MGI Symbol;Acc:MGI:5663580]
ENSMUSG00000075408	6030408B16I	1.007145	6.633402	-2.70107	0.04164	protein_co	RIKEN cDNA 6030408B16 gene [Source:MGI Symbol;Acc:MGI:1924
ENSMUSG00000081223	Gm12247	6.390241	0.61162	3.358421	0.041733	processed_	predicted gene 12247 [Source:MGI Symbol;Acc:MGI:3649489]
ENSMUSG00000028717	Tal1	44.13542	138.8736	-1.65318	0.04183	protein_co	T cell acute lymphocytic leukemia 1 [Source:MGI Symbol;Acc:MGI:
ENSMUSG00000113138	Gm47177	0	3.944431	-4.37152	0.041831	lincRNA	predicted gene, 47177 [Source:MGI Symbol;Acc:MGI:6095962]
ENSMUSG00000103160	C130012C08I	2.955247	14.19729	-2.24294	0.042461	TEC	RIKEN cDNA C130012C08 gene [Source:MGI Symbol;Acc:MGI:2145

ENSMUSG00000111692	Gm49373	3.788072	12.64508	-1.74136	0.042687	protein_co	predicted gene, 49373 [Source:MGI Symbol;Acc:MGI:6121591]
ENSMUSG00000030858	Fam24b	7.463573	1.60247	2.232358	0.043029	protein_co	family with sequence similarity 24 member B [Source:MGI Symbol;
ENSMUSG00000008461	Fut1	4.689425	0.302853	3.78564	0.043052	protein_co	fucosyltransferase 1 [Source:MGI Symbol;Acc:MGI:109375]
ENSMUSG00000039684	Gm5422	0.733276	9.505813	-3.73384	0.043167	transcribec	predicted pseudogene 5422 [Source:MGI Symbol;Acc:MGI:364341
ENSMUSG00000074824	Rslcan18	0.346836	4.547181	-3.61477	0.043281	protein_co	regulator of sex-limitation candidate 18 [Source:MGI Symbol;Acc:M
ENSMUSG00000035238	Kcnk15	6.670889	29.82185	-2.14998	0.043569	protein_co	potassium channel, subfamily K, member 15 [Source:MGI Symbol;A
ENSMUSG00000108547	Gm45186	0	3.725401	-4.2977	0.043587	processed_	predicted gene 45186 [Source:MGI Symbol;Acc:MGI:5753762]
ENSMUSG00000050370	Ch25h	5.022701	17.07898	-1.75732	0.043958	protein_co	cholesterol 25-hydroxylase [Source:MGI Symbol;Acc:MGI:1333869
ENSMUSG00000071691	Gm960	99.45513	61.45929	0.694635	0.044663	protein_co	predicted gene 960 [Source:MGI Symbol;Acc:MGI:2685806]
ENSMUSG00000032259	Drd2	79.90044	283.1917	-1.82486	0.04471	protein_co	dopamine receptor D2 [Source:MGI Symbol;Acc:MGI:94924]
ENSMUSG00000090550	Prlh	9.579751	0.914473	3.3689	0.044949	protein_co	prolactin releasing hormone [Source:MGI Symbol;Acc:MGI:364466
ENSMUSG00000115691	Gm48914	3.553809	14.32954	-2.02425	0.04508	lincRNA	predicted gene, 48914 [Source:MGI Symbol;Acc:MGI:6118221]
ENSMUSG00000104705	4930405N21I	0	3.428461	-4.17764	0.04528	TEC	RIKEN cDNA 4930405N21 gene [Source:MGI Symbol;Acc:MGI:1921
ENSMUSG00000068452	Duox2	0.366638	8.594296	-4.5339	0.046088	protein_co	dual oxidase 2 [Source:MGI Symbol;Acc:MGI:3036280]
ENSMUSG00000024669	Cd5	12.90164	2.205221	2.550219	0.046978	protein_co	CD5 antigen [Source:MGI Symbol;Acc:MGI:88340]
ENSMUSG00000105265	Sox2ot	455.9461	820.1683	-0.84696	0.047466	processed_	SOX2 overlapping transcript (non-protein coding) [Source:MGI Sym
ENSMUSG00000113216	Gm40841	183.5195	276.5336	-0.59026	0.047708	lincRNA	predicted gene, 40841 [Source:MGI Symbol;Acc:MGI:5623726]
ENSMUSG00000031410	Nxf7	1.007145	8.686989	-3.08726	0.047836	protein_co	nuclear RNA export factor 7 [Source:MGI Symbol;Acc:MGI:215934
ENSMUSG00000089737	Gm15688	50.48869	26.28255	0.939063	0.048182	antisense	predicted gene 15688 [Source:MGI Symbol;Acc:MGI:3647549]
ENSMUSG00000045903	Npas4	104.9226	249.0548	-1.24771	0.048239	protein_co	neuronal PAS domain protein 4 [Source:MGI Symbol;Acc:MGI:2664
ENSMUSG00000070084	Mir486	6.17578	0.996764	2.670647	0.048289	miRNA	microRNA 486 [Source:MGI Symbol;Acc:MGI:3619423]
ENSMUSG00000039079	Trhr2	6.155977	21.33341	-1.79266	0.048387	protein_co	thyrotropin releasing hormone receptor 2 [Source:MGI Symbol;Acc
ENSMUSG00000086565	Gm13133	4.227678	0.345477	3.625459	0.048662	antisense	predicted gene 13133 [Source:MGI Symbol;Acc:MGI:3650642]
ENSMUSG00000027210	Meis2	611.5532	1503.817	-1.2981	0.049298	protein_co	Meis homeobox 2 [Source:MGI Symbol;Acc:MGI:108564]
ENSMUSG00000068794	Col28a1	8.58329	23.24158	-1.43371	0.049548	protein_co	collagen, type XXVIII, alpha 1 [Source:MGI Symbol;Acc:MGI:268531
ENSMUSG00000025938	Slco5a1	42.37993	110.6853	-1.38438	0.049603	protein_co	solute carrier organic anion transporter family, member 5A1 [Sourc

HF1G vs STD

gene_id	gene_name	HF1G_fpkr	STD_fpkm	log2FoldCh	pvalue	gene_bioty	gene_description
ENSMUSG00000115625	2900040C04I	113.0136	34.12098	1.732883	0.000624	lincRNA	RIKEN cDNA 2900040C04 gene [Source:MGI Symbol;Acc:MGI:1920
ENSMUSG00000059824	Dbp	1864.062	890.266	1.066155	0.00095	protein_co	D site albumin promoter binding protein [Source:MGI Symbol;Acc:I
ENSMUSG00000026247	Ecel1	191.3658	79.74938	1.265939	0.001222	protein_co	endothelin converting enzyme-like 1 [Source:MGI Symbol;Acc:MGI
ENSMUSG00000036151	Tm6sf2	66.30088	142.0528	-1.09913	0.00143	protein_co	transmembrane 6 superfamily member 2 [Source:MGI Symbol;Acc
ENSMUSG00000061808	Ttr	41233.42	14978.32	1.460955	0.001582	protein_co	transthyretin [Source:MGI Symbol;Acc:MGI:98865]

ENSMUSG00000100241	Slc18a3	54.49013	7.793206	2.817481	0.001586	protein_co	solute carrier family 18 (vesicular monoamine), member 3 [Source:
ENSMUSG00000079845	Xlr4a	14.98909	1.513124	3.279315	0.001942	protein_co	X-linked lymphocyte-regulated 4A [Source:MGI Symbol;Acc:MGI:35
ENSMUSG00000022769	Sdf2l1	236.2118	567.3561	-1.26453	0.002381	protein_co	stromal cell-derived factor 2-like 1 [Source:MGI Symbol;Acc:MGI:2
ENSMUSG00000015652	Steap1	62.88593	21.36309	1.565939	0.003613	protein_co	six transmembrane epithelial antigen of the prostate 1 [Source:MG
ENSMUSG00000057969	Sema3b	244.8598	97.689	1.328395	0.004049	protein_co	sema domain, immunoglobulin domain (Ig), short basic domain, se
ENSMUSG00000025316	Banp	575.0741	1054.056	-0.87418	0.005006	protein_co	BTG3 associated nuclear protein [Source:MGI Symbol;Acc:MGI:188
ENSMUSG00000026051	1500015O10	510.8611	250.8015	1.027821	0.005018	protein_co	RIKEN cDNA 1500015O10 gene [Source:MGI Symbol;Acc:MGI:1926
ENSMUSG00000042328	Hps4	529.7561	865.517	-0.70784	0.005213	protein_co	HPS4, biogenesis of lysosomal organelles complex 3 subunit 2 [Sou
ENSMUSG00000021342	Prl	8.257616	67.15379	-3.02222	0.005425	protein_co	prolactin [Source:MGI Symbol;Acc:MGI:97762]
ENSMUSG00000032575	Manf	775.1208	1390.573	-0.84334	0.00574	protein_co	mesencephalic astrocyte-derived neurotrophic factor [Source:MGI
ENSMUSG00000067455	Hist1h4j	3.157232	15.2114	-2.27841	0.006979	protein_co	histone cluster 1, H4j [Source:MGI Symbol;Acc:MGI:2448436]
ENSMUSG00000022949	Clic6	644.9482	350.5361	0.880364	0.007902	protein_co	chloride intracellular channel 6 [Source:MGI Symbol;Acc:MGI:2146
ENSMUSG00000038550	Ciart	412.7488	217.3406	0.925705	0.008048	protein_co	circadian associated repressor of transcription [Source:MGI Symbo
ENSMUSG00000071665	Foxr2	30.89568	7.76746	1.985723	0.008158	protein_co	forkhead box R2 [Source:MGI Symbol;Acc:MGI:3511682]
ENSMUSG00000002835	Chaf1a	97.46879	51.21988	0.926813	0.008252	protein_co	chromatin assembly factor 1, subunit A (p150) [Source:MGI Symbo
ENSMUSG00000043872	Zmym1	199.0976	440.9983	-1.14827	0.008341	protein_co	zinc finger, MYM domain containing 1 [Source:MGI Symbol;Acc:MG
ENSMUSG00000029847	Slc23a4	18.88338	4.553414	2.031231	0.008393	protein_co	solute carrier family 23 member 4 [Source:MGI Symbol;Acc:MGI:1
ENSMUSG00000001827	Folr1	201.0514	64.04013	1.651109	0.008485	protein_co	folate receptor 1 (adult) [Source:MGI Symbol;Acc:MGI:95568]
ENSMUSG00000020681	Ace	780.6455	411.9943	0.922619	0.008693	protein_co	angiotensin I converting enzyme (peptidyl-dipeptidase A) 1 [Source
ENSMUSG00000022425	Enpp2	10320.49	4982.406	1.050661	0.008872	protein_co	ectonucleotide pyrophosphatase/phosphodiesterase 2 [Source:MG
ENSMUSG00000026864	Hspa5	4112.785	7163.086	-0.80048	0.00952	protein_co	heat shock protein 5 [Source:MGI Symbol;Acc:MGI:95835]
ENSMUSG00000003477	Inmt	39.04553	14.9581	1.382643	0.010194	protein_co	indolethylamine N-methyltransferase [Source:MGI Symbol;Acc:MG
ENSMUSG00000105504	Gbp5	99.24071	34.21311	1.542633	0.010418	protein_co	guanylate binding protein 5 [Source:MGI Symbol;Acc:MGI:2429943
ENSMUSG00000040856	Dlk1	335.667	164.6729	1.02929	0.010473	protein_co	delta like non-canonical Notch ligand 1 [Source:MGI Symbol;Acc:M
ENSMUSG00000024427	Spry4	196.8471	370.2945	-0.91259	0.010511	protein_co	sprouty RTK signaling antagonist 4 [Source:MGI Symbol;Acc:MGI:1
ENSMUSG00000056648	Hoxb8	28.91652	2.267204	3.687411	0.011206	protein_co	homeobox B8 [Source:MGI Symbol;Acc:MGI:96189]
ENSMUSG00000045930	Clec14a	92.16319	46.0174	0.999493	0.011685	protein_co	C-type lectin domain family 14, member a [Source:MGI Symbol;Acc
ENSMUSG00000049580	Tsku	73.08161	35.6873	1.032397	0.012068	protein_co	tsukushi, small leucine rich proteoglycan [Source:MGI Symbol;Acc:
ENSMUSG00000023272	Creld2	347.9534	607.6087	-0.80416	0.012395	protein_co	cysteine-rich with EGF-like domains 2 [Source:MGI Symbol;Acc:MG
ENSMUSG00000028270	Gbp2	114.3302	42.82822	1.418072	0.01268	protein_co	guanylate binding protein 2 [Source:MGI Symbol;Acc:MGI:102772]
ENSMUSG00000027800	Tm4sf1	302.7151	189.3882	0.675893	0.013079	protein_co	transmembrane 4 superfamily member 1 [Source:MGI Symbol;Acc:
ENSMUSG00000047230	Cldn2	120.2221	52.52655	1.197328	0.013567	protein_co	claudin 2 [Source:MGI Symbol;Acc:MGI:1276110]
ENSMUSG00000044285	Gm1821	273.2799	130.0052	1.074561	0.014738	transcribe	predicted gene 1821 [Source:MGI Symbol;Acc:MGI:3037679]
ENSMUSG00000074817	Papolb	1.692014	10.40468	-2.59847	0.01542	protein_co	poly (A) polymerase beta (testis specific) [Source:MGI Symbol;Acc:

ENSMUSG00000039323	Igfbp2	1353.389	674.9372	1.003916	0.015616	protein_co	insulin-like growth factor binding protein 2 [Source:MGI Symbol;Ac
ENSMUSG00000035711	Dok3	251.0038	496.9964	-0.98517	0.015642	protein_co	docking protein 3 [Source:MGI Symbol;Acc:MGI:1351490]
ENSMUSG00000059201	Lep	14.15607	1.065305	3.811329	0.015742	protein_co	leptin [Source:MGI Symbol;Acc:MGI:104663]
ENSMUSG00000054072	Iigp1	75.35629	25.43153	1.571959	0.015839	protein_co	interferon inducible GTPase 1 [Source:MGI Symbol;Acc:MGI:19262
ENSMUSG00000069588	Gm11733	6.356638	0.282266	4.228586	0.016446	protein_co	predicted gene 11733 [Source:MGI Symbol;Acc:MGI:3650737]
ENSMUSG00000088791	Gm22542	0	4.903552	-4.66816	0.016532	snRNA	predicted gene, 22542 [Source:MGI Symbol;Acc:MGI:5452319]
ENSMUSG00000050926	Dcaf12l2	21.60486	5.545008	1.94982	0.01708	protein_co	DDB1 and CUL4 associated factor 12-like 2 [Source:MGI Symbol;Ac
ENSMUSG00000020484	Xbp1	1488.958	2584.361	-0.79557	0.017189	protein_co	X-box binding protein 1 [Source:MGI Symbol;Acc:MGI:98970]
ENSMUSG00000073421	H2-Ab1	91.66292	35.4109	1.375558	0.017272	protein_co	histocompatibility 2, class II antigen A, beta 1 [Source:MGI Symbol;
ENSMUSG00000041809	Efhc1	65.73068	30.9154	1.094621	0.017608	protein_co	EF-hand domain (C-terminal) containing 1 [Source:MGI Symbol;Acc
ENSMUSG00000024610	Cd74	260.7667	91.81485	1.506793	0.018037	protein_co	CD74 antigen (invariant polypeptide of major histocompatibility co
ENSMUSG00000032744	Heyl	102.7886	53.72959	0.939556	0.018222	protein_co	hairy/enhancer-of-split related with YRPW motif-like [Source:MGI S
ENSMUSG00000036198	Arhgap36	51.08975	19.66305	1.387778	0.018231	protein_co	Rho GTPase activating protein 36 [Source:MGI Symbol;Acc:MGI:19
ENSMUSG00000062515	Fabp4	40.51973	7.325505	2.491554	0.018301	protein_co	fatty acid binding protein 4, adipocyte [Source:MGI Symbol;Acc:MG
ENSMUSG00000104093	A330015K06I	62.43283	32.66517	0.937698	0.019356	antisense	RIKEN cDNA A330015K06 gene [Source:MGI Symbol;Acc:MGI:2443
ENSMUSG00000038236	Hoxa7	4.7949	0	4.788616	0.019619	protein_co	homeobox A7 [Source:MGI Symbol;Acc:MGI:96179]
ENSMUSG00000046714	Foxc2	91.70169	37.53652	1.289875	0.02004	protein_co	forkhead box C2 [Source:MGI Symbol;Acc:MGI:1347481]
ENSMUSG00000109353	Gm45183	0.350489	6.818868	-4.19098	0.020135	TEC	predicted gene 45183 [Source:MGI Symbol;Acc:MGI:5753759]
ENSMUSG00000036718	Micall2	72.33612	34.55911	1.065906	0.020884	protein_co	MICAL-like 2 [Source:MGI Symbol;Acc:MGI:2444818]
ENSMUSG00000079363	Gbp4	168.238	42.07419	2.002804	0.021358	protein_co	guanylate binding protein 4 [Source:MGI Symbol;Acc:MGI:97072]
ENSMUSG00000024907	Gal	60.96586	13.15626	2.210371	0.021518	protein_co	galanin [Source:MGI Symbol;Acc:MGI:95637]
ENSMUSG00000050211	Pla2g4e	273.155	142.3008	0.941509	0.02155	protein_co	phospholipase A2, group IVE [Source:MGI Symbol;Acc:MGI:191914
ENSMUSG00000035694	Caps2	12.16692	2.680224	2.225112	0.021738	protein_co	calcyphosphine 2 [Source:MGI Symbol;Acc:MGI:2441980]
ENSMUSG00000018822	Sfrp5	130.7493	50.50822	1.373731	0.022064	protein_co	secreted frizzled-related sequence protein 5 [Source:MGI Symbol;A
ENSMUSG00000004341	Gpx6	6.085335	0.311225	4.173374	0.022177	protein_co	glutathione peroxidase 6 [Source:MGI Symbol;Acc:MGI:1922762]
ENSMUSG00000083226	Gm7831	7.674246	0.919633	3.042371	0.022184	processed_	predicted gene 7831 [Source:MGI Symbol;Acc:MGI:3643841]
ENSMUSG00000112035	Gm49335	31.99032	116.3075	-1.86009	0.022568	lincRNA	predicted gene, 49335 [Source:MGI Symbol;Acc:MGI:6121522]
ENSMUSG00000036169	Sostdc1	162.2524	58.68644	1.469894	0.022994	protein_co	sclerostin domain containing 1 [Source:MGI Symbol;Acc:MGI:1913
ENSMUSG00000073409	H2-Q6	127.0446	36.75027	1.791136	0.02321	protein_co	histocompatibility 2, Q region locus 6 [Source:MGI Symbol;Acc:MG
ENSMUSG00000003484	Cyp4f18	16.10056	4.474739	1.855336	0.0233	protein_co	cytochrome P450, family 4, subfamily f, polypeptide 18 [Source:MG
ENSMUSG00000078853	Igtp	136.6656	55.18068	1.309461	0.023415	protein_co	interferon gamma induced GTPase [Source:MGI Symbol;Acc:MGI:1
ENSMUSG00000037086	Prr32	53.31863	17.29055	1.626571	0.024519	protein_co	proline rich 32 [Source:MGI Symbol;Acc:MGI:1916050]
ENSMUSG00000079737	3110001I22R	20.95589	42.29849	-1.01759	0.025076	protein_co	RIKEN cDNA 3110001I22 gene [Source:MGI Symbol;Acc:MGI:19138
ENSMUSG00000038112	AW551984	179.9094	98.81421	0.86624	0.025263	protein_co	expressed sequence AW551984 [Source:MGI Symbol;Acc:MGI:214

ENSMUSG00000106928	Gm43860	29.43705	62.92885	-1.0964	0.025678	TEC	predicted gene 43860 [Source:MGI Symbol;Acc:MGI:5663997]
ENSMUSG00000023043	Krt18	79.02818	28.81349	1.460584	0.025846	protein_co	keratin 18 [Source:MGI Symbol;Acc:MGI:96692]
ENSMUSG00000086794	Gm11642	23.84465	9.58772	1.325453	0.026821	processed_	predicted gene 11642 [Source:MGI Symbol;Acc:MGI:3651889]
ENSMUSG00000093865	Lrit3	5.371688	0.355102	3.982925	0.027067	protein_co	leucine-rich repeat, immunoglobulin-like and transmembrane dom
ENSMUSG00000089817	Gm7162	18.47346	72.67781	-1.96974	0.027223	processed_	predicted gene 7162 [Source:MGI Symbol;Acc:MGI:3645097]
ENSMUSG00000023945	Slc5a7	40.66311	12.85735	1.676166	0.027329	protein_co	solute carrier family 5 (choline transporter), member 7 [Source:MG
ENSMUSG00000036594	H2-Aa	84.25764	33.83811	1.319684	0.028162	protein_co	histocompatibility 2, class II antigen A, alpha [Source:MGI Symbol;A
ENSMUSG00000031727	Pmfbp1	20.01737	5.808268	1.802091	0.028894	protein_co	polyamine modulated factor 1 binding protein 1 [Source:MGI Symb
ENSMUSG00000083332	Gm7599	12.9705	3.79437	1.776363	0.028897	processed_	predicted gene 7599 [Source:MGI Symbol;Acc:MGI:3644309]
ENSMUSG00000037921	Ddx60	29.579	13.02786	1.189107	0.029481	protein_co	DEAD (Asp-Glu-Ala-Asp) box polypeptide 60 [Source:MGI Symbol;A
ENSMUSG00000092574	2810047C21F	5.082127	0.282266	3.913998	0.029757	transcribe	RIKEN cDNA 2810047C21 gene 1 [Source:MGI Symbol;Acc:MGI:191
ENSMUSG00000004105	Angptl2	126.4996	73.48448	0.786366	0.030008	protein_co	angiopoietin-like 2 [Source:MGI Symbol;Acc:MGI:1347002]
ENSMUSG00000035929	H2-Q4	249.5752	98.48074	1.342761	0.030657	protein_co	histocompatibility 2, Q region locus 4 [Source:MGI Symbol;Acc:MG
ENSMUSG00000064364	mt-Th	159.9078	72.92021	1.134056	0.030692	Mt_tRNA	mitochondrially encoded tRNA histidine [Source:MGI Symbol;Acc:M
ENSMUSG00000028820	Sfpq	2476.42	3794.515	-0.61566	0.030742	protein_co	splicing factor proline/glutamine rich (polypyrimidine tract binding
ENSMUSG00000105012	Gm42813	6.918922	0.919633	2.894927	0.031	lincRNA	predicted gene 42813 [Source:MGI Symbol;Acc:MGI:5662950]
ENSMUSG00000030218	Mgp	589.8307	336.7813	0.80871	0.032012	protein_co	matrix Gla protein [Source:MGI Symbol;Acc:MGI:96976]
ENSMUSG00000002980	Bcam	521.9116	318.5688	0.712538	0.03261	protein_co	basal cell adhesion molecule [Source:MGI Symbol;Acc:MGI:192994
ENSMUSG00000020713	Gh	4.958434	37.11621	-2.89412	0.033466	protein_co	growth hormone [Source:MGI Symbol;Acc:MGI:95707]
ENSMUSG00000022613	Miox	7.221648	0.282266	4.4211	0.034314	protein_co	myo-inositol oxygenase [Source:MGI Symbol;Acc:MGI:1891725]
ENSMUSG00000068323	Slc4a5	240.8517	97.94618	1.300381	0.034902	protein_co	solute carrier family 4, sodium bicarbonate cotransporter, member
ENSMUSG00000023484	Prph	116.7984	46.27716	1.336093	0.03491	protein_co	peripherin [Source:MGI Symbol;Acc:MGI:97774]
ENSMUSG00000108227	4933412L11F	7.573972	0.710203	3.505799	0.035109	TEC	RIKEN cDNA 4933412L11 gene [Source:MGI Symbol;Acc:MGI:1921
ENSMUSG00000074925	Ptar1	30.62112	86.38108	-1.49424	0.035707	protein_co	protein prenyltransferase alpha subunit repeat containing 1 [Sourc
ENSMUSG00000038567	Cyp24a1	3.933643	0	4.496664	0.035884	protein_co	cytochrome P450, family 24, subfamily a, polypeptide 1 [Source:M
ENSMUSG00000065037	Rn7sk	40.52157	76.96611	-0.92836	0.036162	misc_RNA	RNA, 7SK, nuclear [Source:MGI Symbol;Acc:MGI:103186]
ENSMUSG00000026979	Psd4	6.707127	19.58435	-1.55613	0.036183	protein_co	pleckstrin and Sec7 domain containing 4 [Source:MGI Symbol;Acc:I
ENSMUSG00000022878	Adipoq	8.487242	1.065305	3.082978	0.036186	protein_co	adiponectin, C1Q and collagen domain containing [Source:MGI Syn
ENSMUSG00000031344	Gabrq	72.90407	33.81501	1.115391	0.036234	protein_co	gamma-aminobutyric acid (GABA) A receptor, subunit theta [Sourc
ENSMUSG00000027274	Mkks	175.5037	270.5584	-0.62571	0.036243	protein_co	McKusick-Kaufman syndrome [Source:MGI Symbol;Acc:MGI:18918
ENSMUSG00000038537	Mc3r	6.559517	0.948593	2.798997	0.03672	protein_co	melanocortin 3 receptor [Source:MGI Symbol;Acc:MGI:96929]
ENSMUSG00000107698	Gm44430	9.024686	22.83582	-1.34276	0.036885	TEC	predicted gene, 44430 [Source:MGI Symbol;Acc:MGI:5690822]
ENSMUSG00000103952	Gm37268	1.368698	7.778289	-2.48616	0.037183	TEC	predicted gene, 37268 [Source:MGI Symbol;Acc:MGI:5610496]
ENSMUSG00000111013	Gm32468	8.79789	1.897185	2.215979	0.037288	lincRNA	predicted gene, 32468 [Source:MGI Symbol;Acc:MGI:5591627]

ENSMUSG00000009734	Pou6f2	19.34313	5.872026	1.7181	0.037436	protein_co	POU domain, class 6, transcription factor 2 [Source:MGI Symbol;Ac
ENSMUSG00000031738	Irx6	16.07105	4.510413	1.816742	0.03805	protein_co	Iroquois homeobox 6 [Source:MGI Symbol;Acc:MGI:1927642]
ENSMUSG00000032454	Rbp2	6.553431	0.875757	2.845241	0.038496	protein_co	retinol binding protein 2, cellular [Source:MGI Symbol;Acc:MGI:978
ENSMUSG00000022512	Cldn1	83.56159	33.3321	1.328759	0.038607	protein_co	claudin 1 [Source:MGI Symbol;Acc:MGI:1276109]
ENSMUSG00000022056	Adam7	6.281631	0	5.176216	0.038624	protein_co	a disintegrin and metallopeptidase domain 7 [Source:MGI Symbol;A
ENSMUSG00000079065	BC005561	77.76681	164.7106	-1.08345	0.038679	protein_co	cDNA sequence BC005561 [Source:MGI Symbol;Acc:MGI:3040669]
ENSMUSG00000029219	Slc10a4	45.05643	16.96207	1.42105	0.03947	protein_co	solute carrier family 10 (sodium/bile acid cotransporter family), me
ENSMUSG00000074643	Cpne1	2.483426	10.68198	-2.11203	0.039609	protein_co	copine I [Source:MGI Symbol;Acc:MGI:2386621]
ENSMUSG00000101450	Gm28941	4.918593	19.26906	-1.96755	0.04019	sense_intr	predicted gene 28941 [Source:MGI Symbol;Acc:MGI:5579647]
ENSMUSG00000097336	Fendrr	38.51042	19.21112	1.005799	0.040324	processed_	Foxf1 adjacent non-coding developmental regulatory RNA [Source:
ENSMUSG00000021032	Ngb	118.7104	57.97088	1.033555	0.040772	protein_co	neuroglobin [Source:MGI Symbol;Acc:MGI:2151886]
ENSMUSG00000092528	Nlrp1c-ps	3.528808	0	4.341517	0.041033	transcribe	NLR family, pyrin domain containing 1C, pseudogene [Source:MGI S
ENSMUSG00000034918	Cdhr2	14.75295	4.611333	1.663499	0.041099	protein_co	cadherin-related family member 2 [Source:MGI Symbol;Acc:MGI:2
ENSMUSG00000030307	Slc6a11	2617.63	1706.925	0.617078	0.04112	protein_co	solute carrier family 6 (neurotransmitter transporter, GABA), mem
ENSMUSG00000026579	F5	195.0959	83.66431	1.22329	0.041229	protein_co	coagulation factor V [Source:MGI Symbol;Acc:MGI:88382]
ENSMUSG00000103800	Pcdha8	5.861369	19.74172	-1.74653	0.0415	protein_co	protocadherin alpha 8 [Source:MGI Symbol;Acc:MGI:2681879]
ENSMUSG00000097912	6330403N20	7.206149	1.259818	2.522333	0.041882	lincRNA	RIKEN cDNA 6330403N20 gene [Source:MGI Symbol;Acc:MGI:1917
ENSMUSG00000056174	Col8a2	245.2786	120.0294	1.032695	0.042012	protein_co	collagen, type VIII, alpha 2 [Source:MGI Symbol;Acc:MGI:88464]
ENSMUSG00000026686	Lmx1a	42.9324	15.95468	1.43944	0.042195	protein_co	LIM homeobox transcription factor 1 alpha [Source:MGI Symbol;Ac
ENSMUSG00000079436	Kcnj13	87.17851	42.41496	1.044615	0.042346	protein_co	potassium inwardly-rectifying channel, subfamily J, member 13 [So
ENSMUSG00000098202	B830012L14	1.74636	9.212737	-2.39314	0.042356	lincRNA	RIKEN cDNA B830012L14 gene [Source:MGI Symbol;Acc:MGI:2443
ENSMUSG00000059430	Actg2	9.239737	1.158022	2.948644	0.042403	protein_co	actin, gamma 2, smooth muscle, enteric [Source:MGI Symbol;Acc:M
ENSMUSG00000044951	Mylk4	7.111962	1.230858	2.510292	0.042438	protein_co	myosin light chain kinase family, member 4 [Source:MGI Symbol;A
ENSMUSG00000042857	Gm9776	86.44002	50.41025	0.777906	0.042454	bidirection	predicted gene 9776 [Source:MGI Symbol;Acc:MGI:3641792]
ENSMUSG00000060586	H2-Eb1	91.76736	46.87739	0.972548	0.043614	protein_co	histocompatibility 2, class II antigen E beta [Source:MGI Symbol;Ac
ENSMUSG00000021187	Tc2n	20.51447	6.517596	1.66599	0.04372	protein_co	tandem C2 domains, nuclear [Source:MGI Symbol;Acc:MGI:192166
ENSMUSG00000106430	Gm42460	7.24457	0.62245	3.526744	0.044281	TEC	predicted gene 42460 [Source:MGI Symbol;Acc:MGI:5662597]
ENSMUSG00000083826	Gm13039	0	3.555981	-4.20591	0.044726	processed_	predicted gene 13039 [Source:MGI Symbol;Acc:MGI:3649997]
ENSMUSG00000044551	9930012K11	52.19181	27.20704	0.948337	0.044941	protein_co	RIKEN cDNA 9930012K11 gene [Source:MGI Symbol;Acc:MGI:2145
ENSMUSG00000116835	AC165079.1	9.293656	39.14502	-2.07363	0.045029	processed_	ribosomal protein L15 (Rpl15), pseudogene
ENSMUSG00000019906	Lin7a	295.0906	734.5446	-1.31549	0.045375	protein_co	lin-7 homolog A (C. elegans) [Source:MGI Symbol;Acc:MGI:213560
ENSMUSG00000112947	Gm47493	0.673805	6.323958	-3.1919	0.045504	TEC	predicted gene, 47493 [Source:MGI Symbol;Acc:MGI:6096474]
ENSMUSG00000054178	Gm9938	50.19048	125.0312	-1.31583	0.045604	protein_co	predicted gene 9938 [Source:MGI Symbol;Acc:MGI:3641836]
ENSMUSG00000108660	Gm21284	3.492719	0	4.32882	0.045669	antisense	predicted gene, 21284 [Source:MGI Symbol;Acc:MGI:5434639]

ENSMUSG00000030278	Cidec	9.025183	1.332653	2.801331	0.046205	protein_co	cell death-inducing DFFA-like effector c [Source:MGI Symbol;Acc:M
ENSMUSG00000059463	Spag11b	8.411738	0	5.597373	0.046402	protein_co	sperm associated antigen 11B [Source:MGI Symbol;Acc:MGI:36471
ENSMUSG00000076609	Igkc	21.51159	8.377619	1.353005	0.046948	IG_C_gene	immunoglobulin kappa constant [Source:MGI Symbol;Acc:MGI:964
ENSMUSG00000069874	Irgm2	128.498	60.02048	1.100231	0.047058	protein_co	immunity-related GTPase family M member 2 [Source:MGI Symbol
ENSMUSG00000005892	Trh	29.44963	7.35444	1.9919	0.0474	protein_co	thyrotropin releasing hormone [Source:MGI Symbol;Acc:MGI:9882
ENSMUSG00000053375	Atp6v1e2	4.154353	0.311225	3.618754	0.047654	protein_co	ATPase, H+ transporting, lysosomal V1 subunit E2 [Source:MGI Sym
ENSMUSG00000036449	Lcn8	10.40598	0.710203	3.944444	0.047817	protein_co	lipocalin 8 [Source:MGI Symbol;Acc:MGI:2135945]
ENSMUSG00000029322	Plac8	3.501635	0	4.331769	0.048511	protein_co	placenta-specific 8 [Source:MGI Symbol;Acc:MGI:2445289]
ENSMUSG00000097253	Gm26770	20.29709	8.066394	1.325062	0.048737	antisense	predicted gene, 26770 [Source:MGI Symbol;Acc:MGI:5477264]
ENSMUSG00000048772	Tmem53	53.72588	28.34428	0.920663	0.049225	protein_co	transmembrane protein 53 [Source:MGI Symbol;Acc:MGI:1916027
ENSMUSG00000029817	Tra2a	1626.328	2241.118	-0.46247	0.049555	protein_co	transformer 2 alpha [Source:MGI Symbol;Acc:MGI:1933972]
ENSMUSG00000066357	Wdr6	2916.398	2001.764	0.542877	0.04968	protein_co	WD repeat domain 6 [Source:MGI Symbol;Acc:MGI:1930140]
