# Supplementary material for: Effect of Dietary Grapes on Female C57BL6/J Mice Consuming a High-Fat Diet: Behavioral and Genetic Changes
Source: Antioxidants (Basel). 2022 Feb 18;11(2):414. doi: 10.3390/antiox11020414 (PMC8868599; doi:10.3390/antiox11020414)
Supplement: Supplementary file 1 [file antioxidants-11-00414-s001.zip › antioxidants-1558109-SM/S2 - KEGG Pathway analysis.pdf]

S2 - KEGG pathway analysis

Enriched KEGG pathway analysis between HFDvsSTD, HF1GvsHFD and HF1GvsSTD

HFD vs STD

KEGGID	Description	pvalue	geneID	geneName	keggID
mmu04080	Neuroactive ligand-receptor interaction	8.98E-12	ENSMUSG(	Chrna6/Ht	mmu:11440/mmu:15560/mmu:243764/mmu:13489/mmu:14823/mmu:
mmu04612	Antigen processing and presentation	0.00036	ENSMUSG(	H2-Q2/Cd7	mmu:15013/mmu:16149/mmu:14960/mmu:12504/mmu:14961
mmu04020	Calcium signaling pathway	0.001353	ENSMUSG(	Htr2c/Chrr	mmu:15560/mmu:243764/mmu:21336/mmu:54140/mmu:11540/mmu:
mmu04725	Cholinergic synapse	0.003739	ENSMUSG(	Chrna6/Gr	mmu:11440/mmu:14706/mmu:243764/mmu:63993/mmu:213788
mmu04270	Vascular smooth muscle contraction	0.005032	ENSMUSG(	Avpr1a/Ad	mmu:54140/mmu:11540/mmu:11475/mmu:18761/mmu:17540
mmu05330	Allograft rejection	0.005295	ENSMUSG(	H2-Q2/H2-	mmu:15013/mmu:14960/mmu:14961
mmu05332	Graft-versus-host disease	0.006119	ENSMUSG(	H2-Q2/H2-	mmu:15013/mmu:14960/mmu:14961
mmu05320	Autoimmune thyroid disease	0.007493	ENSMUSG(	H2-Q2/H2-	mmu:15013/mmu:14960/mmu:14961
mmu04658	Th1 and Th2 cell differentiation	0.007503	ENSMUSG(	H2-Aa/Cd4	mmu:14960/mmu:12504/mmu:18761/mmu:14961
mmu04640	Hematopoietic cell lineage	0.008153	ENSMUSG(	H2-Aa/Cd4	mmu:14960/mmu:12504/mmu:17380/mmu:14961
mmu04940	Type I diabetes mellitus	0.009034	ENSMUSG(	H2-Q2/H2-	mmu:15013/mmu:14960/mmu:14961
mmu05012	Parkinson disease	0.009323	ENSMUSG(	Th/Slc18a2	mmu:21823/mmu:214084/mmu:13489/mmu:11540/mmu:17709
mmu05310	Asthma	0.009604	ENSMUSG(	H2-Aa/H2-	mmu:14960/mmu:14961
mmu05030	Cocaine addiction	0.010158	ENSMUSG(	Th/Slc18a2	mmu:21823/mmu:214084/mmu:13489
mmu04659	Th17 cell differentiation	0.012835	ENSMUSG(	H2-Aa/Cd4	mmu:14960/mmu:12504/mmu:18761/mmu:14961
mmu05034	Alcoholism	0.014884	ENSMUSG(	Th/Gng4/S	mmu:21823/mmu:14706/mmu:214084/mmu:13489/mmu:11540
mmu05416	Viral myocarditis	0.022011	ENSMUSG(	H2-Q2/H2-	mmu:15013/mmu:14960/mmu:14961
mmu04614	Renin-angiotensin system	0.022744	ENSMUSG(	Ace/Mme	mmu:11421/mmu:17380
mmu04728	Dopaminergic synapse	0.031111	ENSMUSG(	Th/Gng4/S	mmu:21823/mmu:14706/mmu:214084/mmu:13489
mmu04024	cAMP signaling pathway	0.031889	ENSMUSG(	Chrm2/Drd	mmu:243764/mmu:13489/mmu:20609/mmu:333329/mmu:11540

HF1G vs HFD

KEGGID	Description	pvalue	geneID	geneName	keggID
mmu04080	Neuroactive ligand-receptor interaction	8.84E-05	ENSMUSG(	Gh/Chrna6	mmu:14599/mmu:11440/mmu:243764/mmu:14823/mmu:140498/mmu
mmu04725	Cholinergic synapse	0.001296	ENSMUSG(	Fos/Chrna(	mmu:14281/mmu:11440/mmu:243764/mmu:14706
mmu04728	Dopaminergic synapse	0.002131	ENSMUSG(	Fos/Gng4/	mmu:14281/mmu:14706/mmu:214084/mmu:13489
mmu05034	Alcoholism	0.004281	ENSMUSG(	Hist1h4j/G	mmu:319159/mmu:14706/mmu:214084/mmu:13489
mmu05170	Human immunodeficiency virus 1 infectio	0.010511	ENSMUSG(	Fos/Gng4/	mmu:14281/mmu:14706/mmu:110557/mmu:12504
mmu04724	Glutamatergic synapse	0.012275	ENSMUSG(	Gng4/Grm	mmu:14706/mmu:14823/mmu:329502

mmu04726	Serotonergic synapse	0.015821	ENSMUSG(	Gng4/Slc18	mmu:14706/mmu:214084/mmu:329502
mmu04926	Relaxin signaling pathway	0.017239	ENSMUSG(	Fos/Gng4/	mmu:14281/mmu:14706/mmu:140498
mmu05030	Cocaine addiction	0.018522	ENSMUSG(	Slc18a2/Dr	mmu:214084/mmu:13489
mmu04514	Cell adhesion molecules (CAMs)	0.022332	ENSMUSG(	H2-Q6/Cd4	mmu:110557/mmu:12504/mmu:12511
mmu05031	Amphetamine addiction	0.031707	ENSMUSG(	Fos/Slc18a	mmu:14281/mmu:214084
mmu04612	Antigen processing and presentation	0.035444	ENSMUSG(	H2-Q6/Cd4	mmu:110557/mmu:12504
mmu05167	Kaposi sarcoma-associated herpesvirus inf	0.038593	ENSMUSG(	Fos/Gng4/	mmu:14281/mmu:14706/mmu:110557
mmu05168	Herpes simplex infection	0.040307	ENSMUSG(	Fos/H2-Q6	mmu:14281/mmu:110557/mmu:170722
mmu04020	Calcium signaling pathway	0.043249	ENSMUSG(	Erbb3/Chrı	mmu:13867/mmu:243764/mmu:170732
mmu04024	cAMP signaling pathway	0.049453	ENSMUSG(	Fos/Chrm2	mmu:14281/mmu:243764/mmu:13489

HF1G vs STD

KEGGID	Description	pvalue	geneID	geneName	keggID
mmu04612	Antigen processing and presentation	6.59E-07	ENSMUSG(	Hspa5/H2-	mmu:14828/mmu:14961/mmu:16149/mmu:110557/mmu:14960/mmu:
mmu05330	Allograft rejection	1.13E-05	ENSMUSG(	H2-Ab1/H2	mmu:14961/mmu:110557/mmu:14960/mmu:15015/mmu:14969
mmu05332	Graft-versus-host disease	1.28E-05	ENSMUSG(	H2-Ab1/H2	mmu:14961/mmu:110557/mmu:14960/mmu:15015/mmu:14969
mmu05320	Autoimmune thyroid disease	1.82E-05	ENSMUSG(	H2-Ab1/H2	mmu:14961/mmu:110557/mmu:14960/mmu:15015/mmu:14969
mmu04940	Type I diabetes mellitus	2.53E-05	ENSMUSG(	H2-Ab1/H2	mmu:14961/mmu:110557/mmu:14960/mmu:15015/mmu:14969
mmu04514	Cell adhesion molecules (CAMs)	8.05E-05	ENSMUSG(	Cldn2/H2-/	mmu:12738/mmu:14961/mmu:110557/mmu:14960/mmu:15015/mmu:
mmu05416	Viral myocarditis	0.000114	ENSMUSG(	H2-Ab1/H2	mmu:14961/mmu:110557/mmu:14960/mmu:15015/mmu:14969
mmu05310	Asthma	0.000218	ENSMUSG(	H2-Ab1/H2	mmu:14961/mmu:14960/mmu:14969
mmu04145	Phagosome	0.000928	ENSMUSG(	H2-Ab1/H2	mmu:14961/mmu:110557/mmu:14960/mmu:15015/mmu:14969/mmu:
mmu05168	Herpes simplex infection	0.002043	ENSMUSG(	H2-Ab1/Cc	mmu:14961/mmu:16149/mmu:110557/mmu:14960/mmu:15015/mmu:
mmu05323	Rheumatoid arthritis	0.002208	ENSMUSG(	H2-Ab1/H2	mmu:14961/mmu:14960/mmu:14969/mmu:74915
mmu04672	Intestinal immune network for IgA produc	0.00314	ENSMUSG(	H2-Ab1/H2	mmu:14961/mmu:14960/mmu:14969
mmu05150	Staphylococcus aureus infection	0.005338	ENSMUSG(	H2-Ab1/H2	mmu:14961/mmu:14960/mmu:14969
mmu05322	Systemic lupus erythematosus	0.005929	ENSMUSG(	Hist1h4j/H	mmu:319159/mmu:14961/mmu:14960/mmu:14969
mmu05145	Toxoplasmosis	0.00785	ENSMUSG(	H2-Ab1/Igt	mmu:14961/mmu:16145/mmu:14960/mmu:14969
mmu05321	Inflammatory bowel disease (IBD)	0.007866	ENSMUSG(	H2-Ab1/H2	mmu:14961/mmu:14960/mmu:14969
mmu05140	Leishmaniasis	0.012006	ENSMUSG(	H2-Ab1/H2	mmu:14961/mmu:14960/mmu:14969
mmu05166	Human T-cell leukemia virus 1 infection	0.012994	ENSMUSG(	Xbp1/H2-A	mmu:22433/mmu:14961/mmu:110557/mmu:14960/mmu:15015/mmu:
mmu05169	Epstein-Barr virus infection	0.015177	ENSMUSG(	H2-Ab1/H2	mmu:14961/mmu:110557/mmu:14960/mmu:15015/mmu:14969
mmu04918	Thyroid hormone synthesis	0.016562	ENSMUSG(	Ttr/Hspa5/	mmu:22139/mmu:14828/mmu:75512
mmu00590	Arachidonic acid metabolism	0.021965	ENSMUSG(	Pla2g4e/G	mmu:329502/mmu:75512/mmu:72054
